# Newly engineered alumina quantum dot-based nanofluid in enhanced oil recovery at reservoir conditions

**DOI:** 10.1038/s41598-022-12387-y

**Published:** 2022-06-09

**Authors:** Nosrat Izadi, Bahram Nasernejad

**Affiliations:** Department of Chemical Engineering, Amirkabir University of Technology, Tehran, 15875-4413 Iran

**Keywords:** Energy science and technology, Nanoscience and technology

## Abstract

In this work, a newly engineered alumina quantum dot-based nanofluid (α-AQDs; D ~ 4 nm; amorphous solid) and one commercial alumina nanoparticle-based nanofluid (γ-ANPs; D ~ 20 nm; crystalline type) with the capability of strong colloidal dispersion at reservoir conditions, such as, high salinity, divalent ions (Ca^2+^) and high temperature was compared. The main goal of this research was to study the crude oil displacement mechanisms of alumina suspensions as a function of variety in size and particle morphology in aged carbonate rocks. The strong interaction potential between the particles was achieved by the citric acid and a special composition of a carboxylate-sulfonate-based polyelectrolyte polymer as an effective dispersant compound on the surface, leading to a negative particle charges and an additional steric and electrostatic repulsion. Wettability alteration upon exposure to fluids using the contact angle and the Amott cell were performed on saturated carbonate plug samples and rock slices. While, dynamic core displacements were conducted to test the water/nanofluid/oil flow and nanoparticle retention behavior thorough typical pore throats underground the reservoir conditions. The stability results revealed that PE-polymer was able to create a long-term colloidal fluid during 30 days. It was found that mass concentration of nanofluid increased with decreasing in particle size. The optimal amount of particles in aqueous solution was obtained 0.05 wt% for ANPs, increased up to 0.1 wt% for AQDs. Analysis of experiments showed that wettability alteration was the main mechanism during nanofluid injection. Laboratory core-flooding data proved that the enhanced oil recovery due to a less concentration state by ANPs was consistent with AQDs at higher concentrations. In addition, permeability-impairment-behavior study was discussed in terms of possible mineral scale deposition and alumina release on the rock surface. Results showed that a large extent of permeability damage caused by mineral scale (55–59%). Alumina quantum dot-based nanofluids were found a minimum impairment (2–4%) and a significant reduction of ~ 10% in permeability was observed for ANPs-based nanofluid.

## Introduction

The global demand for oil has increased in recent years and the oil primary generation cannot supply this request any more. Almost, the remaining two-third of oil-in-place of reservoirs is prone for enhanced oil recovery (EOR). Of the numerous EOR methods, chemical methods have been considered as the most improvements because of their high efficiency and technical and economical feasibilities. Nanofluids, which are prepared by dispersing of nanoparticles with a dimension between 1 and 100 nm in a host fluid and can be injected into the reservoirs to boost oil production, have been attracted by many researchers in recent decades and majorly categorized into chemical EOR methods^1–7^. Also, some researchers have been interested in mixing of the nanoparticles with other chemicals such as surfactants, polymers, foam and emulsions to move forward both oil field characterization and recovery^8–16^. Among the numerous parameters that influence on the oil recovery efficiency, wettability alteration^17–19^ and interfacial tensions (IFTs)^20,21^ are the most important factors affecting the oil recovery. When new materials, such as nanoparticles have been applied as an additive in the water flooding^5,22–28^, the enhancing oil recovery mechanisms by them have been explored in various investigations. Some these mechanisms include: (1) disjointing pressure gradient that cause separating of oil droplets from the rock surface by creating a wedge-film^29,30^; (2) mobility ratio decreasing and increasing the injected fluid viscosity^31,32^, (3) interfacial tension reduction^33–35^ and (4) wettability alteration to more water-wet conditions^36–40^. However, one of the main challenges for using particles in enhanced oil recovery is that they must be colloidal dispersion at reservoir conditions such as high-temperature and strong saltiness containing divalent ions such as Ca^2+^ and Mg^2+^. Nanofluids with low stability transport in reservoir media may cause harsh damage in porous media^33,41^. Stability of nanofluids has been broadly examined in Ghadimi’work for various thermodynamic conditions progressively in cooling towers and other heat transfer mediums^42^. Ogolo et al.^43^ applied a few kinds of nanoparticles, for example, aluminum oxides, zinc, magnesium, iron, zirconium, nickel and silicon on oil-wetted sand. They showed that using nanofluids would be very effective in oil recovery. They considered effective mechanisms for recovery enhancement were wettability alteration, reduction of interfacial tension, oil viscosity and mobility ratio. Ju et al.^44^ investigated hydrophilic polysilicon nanoparticles for wettability alteration in rock surface. They revealed that oil recovery could clearly be improved by flooding with hydrophilic polysilicon nanoparticles. This work recommended that a nanoparticle concentration between 0.02 to 0.03 wt% was desirable for enhanced oil recovery. Also, the results of Maghzi et al.^38^ showed that the silica nanoparticles caused enhancement of sweep efficiency during water flooding. They used micromodel with five-spot glass which was initially saturated with the heavy oil and water at different values of weight percent. Giraldo et al.^45^ investigated on adjusting the wettability of sandstone centers with initial oil-wettability in the present of alumina-based nanofluids in reservoir. Khosravani et al.^46^ used γ-Al_2_O_3_ nanoparticles with different specific surface areas and produced fluid samples by a simple and economical technique. They investigated the stability of hybrid nano-emulsions in different temperatures, followed by developing a stable emulsion and applying it in oil recovery tests. Also Mohammadi et al.^47^ reported the same results for synthesis of γ-Al_2_O_3_ nanoparticles. They investigated the effect of γ-Al_2_O_3_ nanoparticles on wettability alteration of carbonate reservoir. Khezrnejad et al.^48^ studied the effect of different factors such as nanoparticle type (silica oxide and aluminum oxide), concentration of nanoparticles, pressure, temperature and injection rate on the enhanced oil recovery. They showed that different factors had a significant effect on the viscosity and oil recovery than the type of the nanoparticles. Cao et al.^49^ showed that alumina, silica, and zirconium nanofluids have the highest impact on reducing of the surface contact angle and wettability alteration. The effect of the type and concentration of each of nanoparticles were determined. Also Lu et al.^50^ showed synthesis methods and conditions have a great effect on the nanoparticles properties and stability. They synthesized the γ-Al_2_O_3_ nanostructure by hydrothermal method. Nevertheless, over the past few decades, new nanofluids such as polymer nanoparticles have been widely used in pollution control and medicine^51^ due to its low toxicity and good biocompatibility^52^ which made them suitable for oil reservoir. Furthermore, the surface of polymer nanoparticles could be modified easily especially with carboxyl groups^53^, which made them possible to be stable in high-temperature and high-salinity. Therefore, the polymer-coated nanoparticles were good candidate carriers. Bila et al.^54^ explained the applicability of polymer-coated silica nanoparticles as additives for water flooding improving in water wet reservoirs. They proposed IFT reduction, alteration in the rock surface roughness and wettability to more water-wet and microscopic flow diversion due to clogging of the pores are the main EOR mechanisms in the application of nanoparticles. Moreover, the nanoparticles mobilized residual oil and increased oil recovery up to 9.2% of the OOIP^54^. Zhou et al.^55^ developed a new nanofluid by coating negatively charged polymer nanoparticles with betaine-type zwitterionic surfactant via electrostatic attraction. The nanofluid enhanced stability at high salinities brine containing divalent ions (15% stimulated brine) and high temperatures (80˚C). The capability of the nanofluid for enhanced oil recovery revealed that the total oil recovery by nanofluid was 9.32% higher than that of the brine^55^. Also, Omran et al.^56^ studied the performance of polymer-coated silica NPs for oil recovery on micro-scale at three wettability conditions (water-wet, intermediate-wet and oil-wet) while all laboratory conditions such as flow rate, pore structure, initial oil connectivity and temperature were considered the same for all states. The clusterization efficacy of polymer-coated silica NPs, due to a higher mobilization, smaller remaining oil clusters, and lower connectivity of the residual oil was obtained better. Sagala et al.^57^ synthesized and investigated the performance of various types of nanopyroxenes as neutral pyroxene, half hydroxyl functionalized and fully hydroxyl functionalized hydrophobic pyroxene. The concentration of these nanofluids were kept constant at 0.005 wt%. The efficiency of the different pyroxene-based nanofluids has been examined through contact angle, IFT measurements at various temperatures, spontaneous imbibition (SI), and core flooding tests. Oil recovery was increased an additional 10.57% after brine flooding through core flooding tests^57^. Ali et al.^58^ prepared smart-polymeric-nanofluids by dispersing the synthesized TiO_2_/SiO_2_/PAMNCs in smart water with different types and concentrations of dissolved ions. The formulated smart-polymeric-nanofluids were applied to ITF reduction and wettability alteration of carbonate rocks measurements. The highest increase in oil recovery was obtained from 36.0 to 46.5% of the original oil in place (OOIP)^58^. In this study, as a metal oxide-based nanofluid, our selections were limited on aluminum oxide (Al_2_O_3_) which is of interest in various applications especially as an improvement agent for increasing of subsurface oil reserviors^45–47,59–61^. So, in the present work, we synthesized alumina quantum dots (AQDs) with the aim of producing nanostructures with very small size and quantum properties. Then EOR performance of alumina quantum dot-based nanofluids (AQDs; D ~ 4 nm) were investigated and compared with commercial nanoparticles (ANPs; D ~ 20 nm) at reservoir conditions, such as, simultaneous effect of temperature, salinity and divalent ions (Ca^2^) in the presence of carbonate rock. In general, the main objective of this study was to demonstrate the response of the particle size and morphology type of alumina in quantum dot and nanoparticle solid on colloidal stability and then to gain insight into the underlying EOR potential of them including wettability alteration through the contact angle measurements and the Amott cell tests and dynamic core displacements. Also, we followed a perfect methodology as in carbonate rock to consider formation damage behavior of particles. We synthesized AQDs according to Nemade’work^62^ and supplied commercial ANPs from Scharlau company. Then, we enhanced negatively charged carboxylated (COO^−^) groups onto alumina surface thorough citric acid and polyelectrolyte polymer (PE) coatings to ensure them remain stable. Experimental results indicated that 0.05 wt% of cit-AQDs powder + 0.05 wt% of PE in SWP, 0.1 wt% of cit-AQDs powder + 0.1 wt% of PE in SWP and 0.05 wt% of cit-ANPs powder + 0.1 wt% of PE in SWP could produce strong fluids with very long-term stability which could change wettability alteration towards more water-wetness, increased additional oil recovery of about 24–38% than brine. Also, the laboratory experiments showed that the formation damage from PE-cit-ANPsswp nanofluid injection even with lower concentration, was severe. The formation damage was reduced significantly when PE-cit-AQDsswp was added to brine.

## Results and discussion

### Synthesis and characterization of alumina nanostructures

**Figure 1:**
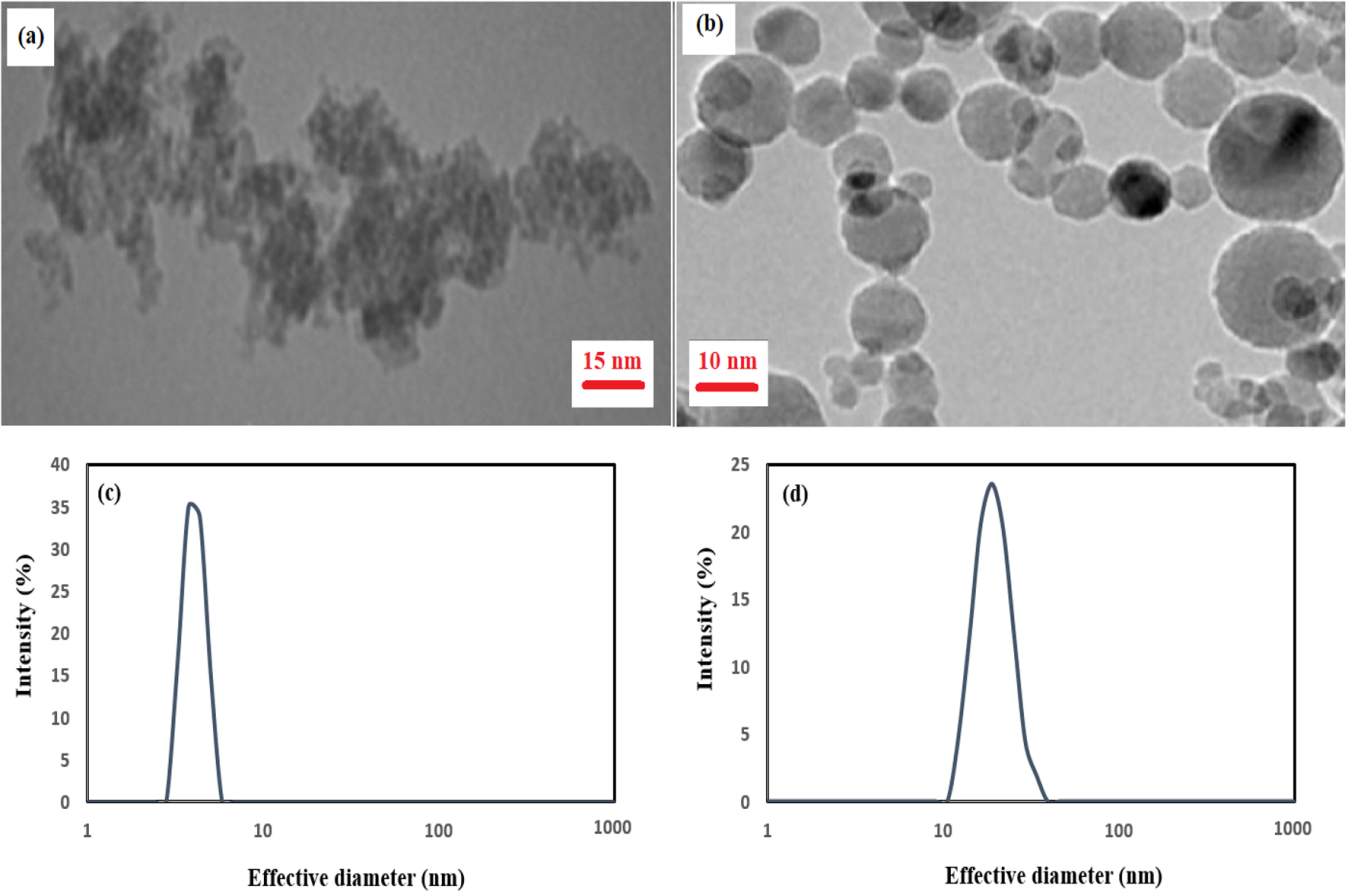
(**a**) TEM micrograph for α-Al_2_O_3_ quantum dots (AQDs), (**b**) TEM image for γ-Al_2_O_3_ nanoparticles (ANPs), (**c**) particle size distribution by DLS for α-Al_2_O_3_ quantum dots, (**d**) particle size distribution by DLS for γ-Al_2_O_3_ nanoparticles.

First, α**-**Al_2_O_3_ quantum dot powder (AQDs) was synthesized by mixing 1 M aluminum nitrate and 1 M hexamethylenetetramine under magnetic stirring at room temperature for 20 min, then centrifuged precipitate was sintered at 200 °C for 36 h according to Nemade’work^62^. Both alumina particles, γ-Al_2_O_3_ nanoparticles powder (ANPs) (< 99.99%, Scharlau company) and synthesized α**-**Al_2_O_3_ quantum dot (AQDs) were characterized by XRD for phase identification, crystallite size and structure determination. (Fig. S1 supporting information) shows XRD patterns of alumina particles. They reveal clearly the presence of Al_2_O_3_ (α and γ) as the aluminum-containing phase detected in samples. (Fig. S1a supporting information), shows the powder X-ray diffractions of α-Al_2_O_3_ quantum dots (AQDs) with planes 012, 104, 110, 113, 024, 116, 124 and 330 of alumina. The XRD pattern of AQDs shows that the peaks are very broadening and this broadening reflects the decreases of crystallite, thus the structures can be considered as amorphous or very fine particles. Based on the Debye–Scherrer equation, the crystallite size of AQDs from diffraction peaks was found about ~ 4 nm. Also, the reflections at 37.31°, 45.34° and 67.43° were found in γ-Al_2_O_3_ nanoparticles (ANPs) with planes (311), (400), (440) of alumina with about ~ 20 nm crystallite size from Debye–Scherrer equation and crystalline structure (Fig. S1b supporting information)_._ Figure 1a and b shows spherical morphology of alumina nanostructures by TEM photographs. The particle size distribution was estimated by measuring the size of total particles of about 100 particles in the TEM image using the image microstructure measurement program and dynamic light scattering analyzer. As seen in Fig. 1c and d, size distribution of particles revealed an average diameter of 4 nm for α-Al_2_O_3_ quantum dots (AQDs) and 20 nm for γ-Al_2_O_3_ nanoparticles (ANPs). The results of ASAP analysis for the total BET surface area and pore volume have been summarized in (Table S3 supporting information). In both samples, there are significant changes in surface area and pore volume. For example, compared with ANPs sample, the total BET surface area of AQDs sample has been increased, where it resulted in 19.7% and 58.2% growth for the total BET surface area and pore volume, respectively. In order to detect properties of quantum dots in synthesized AQDs sample, a fluorescence measurement was made from 300 to 500 nm by PL analyzer and presented in (Fig. S2 supporting information). The peak value of emission was observed at ~ 387 nm which is assigned to the presence of defects.

### Preparation of alumina dispersion

One of the main challenges for using particles in enhanced oil recovery is that they must be colloidal dispersion at reservoir conditions such as high-temperature and strong saltiness containing divalent ions such as Ca^2+^. In this section, aggregate behavior of particle suspensions (AQDs and ANPs) in brines (SWP, SB1, SB2, SB3 and SB4) at 25 and 90 °C was examined. Particle concentrations of 100 to 1200 ppm were chosen with enough focus over the entire stability conditions. From thermodynamics and experimental visual results, Al_2_O_3_ without adding any stabilizer is known to be insoluble in ionic strength media at room temperature. To enhance the dispersion in saline phase and high temperature simultaneously, we created and enhanced negatively charged carboxylated (COO^−^) groups to surface of Al_2_O_3_ by citric acid and polyelectrolyte polymer (PEs) coatings to ensure them remain suspended that aggravate steric, electrostatic or electrostric stability. Citric acid and polyelectrolyte polymer with carboxylated-based chains (acryl amido-sulfonic acid/acrylic acid/acryl amido) dispersants were identified as dual candidate for stabilization. They are able to adsorb on alumina surface and can dissociate and act as surface-charge modifiers. The coating nanoparticles using citric acid and polyelectrolytes (PEs) has been well established in many researchers^63–69^. These additives cause a change of the surface charge resulting in a change of the double layer repulsion by adsorption on the particle surface^68,70^. According to Chapel’s work^66^, long-term colloidal stability can occur when nanoparticles are coated with an organic layer such as polyelectrolyte polymers. The reasons of stabilization behavior of nanoparticles by polymers are well understood^66^: (i) polymers can act as a steric barrier against flocculation through a brush of stretched chains. (ii) When the polymer is bearing charges along its backbone, as in polyelectrolytes, the organic layer imparts an additional electrostatic repulsion between NPs^66^. Steric and electrostatic repulsion that is often referred to electrostric interaction is created by PEs-polymers.

### Citrate-coated-Al_2_O_3_

In this study, initially, citrate-coated-Al_2_O_3_ (AQDs and ANPs, at pH 7.1) were prepared using the method described by Hidber^68^. To obtain the adsorption curves, suspensions of 1.5 g Al_2_O_3_ in 20 ml citric acid solution and inert electrolyte KNO_3_ (0.1 N) were prepared with different amounts of citric acid at pH value of 7.1. The water used was deionized grade and the pH was adjusted with KOH solution (1 N). While adding Al_2_O_3_, the pH was monitored constantly and adjusted. The pH was measured with a glass electrode against a silver/silver chloride electrode (pH meter 86505 AZ instrument). Experimental tubes were purged with N_2_ gas to prevent formation of metal carbonates in solution. Then, the tubes were crimp sealed and put into a mechanical shaker for 72 h at 25 °C and then centrifuged. After centrifugation, the citric acid concentration in the supernatant was determined using a liquid chromatography HPLC (model 600E) instrument equipped with a diode array UV–Vis detector and compared to blank solution (minus adsorbent). The difference between the initial and final citric acid concentrations in the aqueous phase corresponds to the amount of dispersant that adsorbed on surface solid. Prior to its use, the alumina was cleaned using soxhlet extraction with distilled water. The two-surface Langmuir isotherms [Eq. (1)] was used in the interpretation of adsorption phenomena^63^. Two-surface Langmuir model assumes that adsorption occurs on two types of surface of contrasting bonding energies. Adsorption behavior of citric acid on alumina surface (AQDs and ANPs) has been shown in (Fig. S3 supporting information). The maximum adsorbed amount of citric acid was calculated using Eq. (1) from this model^63^ with curve fitting tool box of MATLAB software.

1 $$\left\{{\mathrm{cit}}_{\mathrm{ad}}\right\}=\frac{{\left\{{\mathrm{cit}}_{\mathrm{ad}}\right\}}_{\mathrm{max},1}{\mathrm{K}}_{1}\left[cit\right]}{1+{\mathrm{K}}_{1}\left[cit\right]}+\frac{{\left\{{\mathrm{cit}}_{\mathrm{ad}}\right\}}_{\mathrm{max},2}{\mathrm{K}}_{2}\left[cit\right]}{1+{\mathrm{K}}_{2}\left[cit\right]}$$

where “cit_ad_” is the amount of adsorbed citric acid, “cit” is equilibrium concentration of acid citric in the solution, “(cit_ad_)_max1_ and (cit_ad_)_max2_” are called the measured values for the maximum adsorbed amount of citric acid and K_1_ and K_2_ are adsorption/desorption equilibrium constants. The measured values for the maximum adsorbed amount of citric acid ((cit_ad_)_max1_ + (cit_ad_)_max2_) were found 1.22 and 0.79 µmol/m^2^ for AQDs and ANPs, respectively. In general, citric acid is a tricarboxylic acid with pK_a_ = 3.13, pK_a_ = 4.76, pK_a_ = 6.40 that can form different species (H_3_Cit, H_2_Cit^-^, HCit^2−^ and Cit^3−^) depending on solution pH. Citric acid molecule with three carboxyl groups (COO^-^) and one hydroxyl group, once deprotonated, the three carboxyl groups are charge carriers and enable it as an adsorbent and can form a strongly coordinating complex on the surface solid. The adsorption of citrate on alumina surface dependent on the pH value of the suspension and the concentration of the citric acid. The general trend of citric acid adsorption on alumina surface is a decrease with increasing solution pH, mainly because the electrostatic attraction between the positively charged alumina surface and the citrate anion decreases^68^. The maximum amount of citric acid adsorbed on alumina surface happens at pH 3 and decreases considerably at alkaline media^68^. Acid dissociation constant (pK_a_) values of citric acid vary between 3 and 6 at pH 3 to pH 8^71^. In this work, we modified alumina surface with citric acid at pH 7.1. At this pH value, citric acid exists in the form of protonated species according to Eq. (2) with acid dissociation constant (pK_a_) value of 6.396 at ionic strength, I = 0^72^.

2 $${\mathrm{HCit}}^{2-}\leftrightarrow {\mathrm{Cit}}^{3-}+{\mathrm{H}}^{+} \quad \quad \text{Fully} \, \text{deprotonated}$$

Citrate-alumina surface complex was also investigated with FT-IR technique. Figure S4 (supporting information) shows the FT-IR spectra of citrate-alumina powder. In solution, citric acid is fully deprotonated at pH 7.1. The fully deprotonated citrate ion has prominent absorption bands around 1570 and 1391 cm^−1^ and a weak band around 1280 cm^−1^. The two prominent bands, 1570 and 1391 cm^−1^, are attributed to the asymmetric and symmetric stretching motions of the carboxylate group, respectively^68,73–75^. The weaker band at 1280 cm^−1^ is related to coupled stretches and bends of the carboxylate group. The FT-IR spectrum in (Fig. S4 supporting information) shows that acid citric has been adsorbed on alumina in both samples. The asymmetric *v*_*a*_ (COO^−^) and symmetric *v*_*s*_ (COO^−^) stretching vibration of carboxylate groups is observed in around 1572 and 1395 cm^−1^ for alumina samples that agree well with the Hidber’work^68^.

The phase behavior of citrate-alumina (100 to 1200 ppm) was studied in SWP at 25 and 90 °C. Sedimentation experiments were conducted with a UV–Vis spectrometer as a function of time. At first, a calibration curve had to be made between the light absorbency and the concentration of alumina solution. A calibration curve was made for various concentrations of NP in SWP. Suspensions of 200 ml Al_2_O_3_ were prepared with various concentrations of powder to brine keeping mechanical stirring and ultrasonicating (Ultrasonic Cleanser, 100 W, Equipment Company, Italy) at least for 30 min. Prepared suspensions (1 ml) were placed in UV–Vis instrument immediately and the amount of transmitted light was measured. A linear relationship between the scattered light and alumina concentrations was obtained for calibration plots (Fig. S5 supporting information). Then the samples were allowed to stay overnight to achieve the aggregation just due to gravitational forces and happen steady state media. Supernatant was measured UV–Vis analyzer again. Flocculation plots of fluids have been shown in (Fig. S6 supporting information). In accord with the data in (Fig. S6 supporting information), citrate-coated-AQDs samples were very stable in SWP in only concentrations of 100 to 200 ppm without any flocculation for particles at room temperature. But, a significant transition from the dispersed to the flocculated state corresponded to instability approximately 6 to 40% was observed for concentrations of 300 to 1200 ppm at the same conditions. This phenomenon was repeated around 12 to 90% sedimentation for citrate-coated-ANPs. Sharp instability was seen at T = 90 °C for both samples. However, from the data outlined in (Fig. S6 supporting information), citric acid alone displayed a poor dispersant at concentrated electrolyte solution and elevated temperature. Generally, at low salinities and room temperature, citric acid alone as a small ligand may be utilized to improve electrostatic repulsion^71,76–81^. But at harsh ionic strength, the electrostatic repulsion provided by small ligands becomes insufficient to overcome the attractive Vander Waals forces. At such high salinities, polyelectrolyte polymers are often required to prevent flocculation^82–84^. Combination of electrostatic and steric repulsion is happened with PEs polymers when are present on the particle surface. Therefore, to further enhance the dispersion of nanostructures, we selected PE polymer (acrylamido-sulfonic acid/acrylic acid/acryl amido) to overcome the aggregation problems in brine at 90 °C.

### PE-citrate-alumina complex

Acrylamido-sulfonic acid/acrylic acid/ acryl amido polymer (molecular weight of 52,000 g/mol) with rich carboxylate and sulfonate groups is very soluble in injected waters (SWP, SB1, SB2, SB3 and SB4) without any changes in pH-value. The optimal polymer content required to achieve enough dispersion was obtained in a stability rout. To prepare stock fluids, polyelectrolyte polymer in various amounts (optimal twofold greater than cit-ANPs weight and equal cit-AQDs weight; Table 1) was dissolved in SWP (200 ml), then the citrate-alumina powder was spread at 25 °C. The stability of particle suspensions was measured under the same conditions for citrate-particles. (Fig. S7 supporting information) clearly demonstrates an acceptable trend in adsorption of polymer on particle surface especially at elevated temperature. These observations (at 90 °C) show that cit-AQDs fluids at different particle values ≤ 1000 ppm were fully colloidal solutions and saturated with C/C_0_ approaching 1. In this condition, cit-ANPs fluids achieved upon ≤ 500 ppm. It is believed that polymer adsorption happens stronger for lower particle size compounds likely due to increase in surface charges through their quantum properties. Also, the data presented in (Fig. S7 supporting information) show that the sulfonic-based polyelectrolytes reversed destabilization of fluids at 90˚C and significantly enhanced the colloidal stability than room temperature. The particles settled out of fluid after overnight (~ 2%) for cit-AQDs ≥ 0.12 wt% and (~ 2–12%) for cit-ANPs ≥ 0.06 wt% solutions at 90 °C. Furthermore, we performed extra stability tests with solutions of 0.05 and 0.1 wt% of cit-AQDs and 0.05 wt% of cit-ANPs in brines of different salinities to investigate the effect of extremely concentrated ionic media such as, high salinity and divalent cations like Ca^2+^ on stability of nanofluids. We prepared solutions by spreading of the citrate-alumina powder (cit-AQDs, 0.05 and 0.1 wt%; cit-ANPs, 0.05 wt%) to rich-polymer brines. Therefore, all synthetic brines (SB1, SB2, SB3 and SB4) had various optimal formulations that different in polymer contents (described in Table 1). Complementary, D_H_ values of coated-particles as a function of time were measured with DLS analyzer at 90 °C for up to 1 month. A quantitative evaluation of the salinity-dependent dispersion behavior of these nanofluids have been listed in (Fig. S8 supporting information) and Table 2. A deep colloidal dispersion did exist between the all colloidal species in brines of (SWP; 4.1 wt% salinity + 0.05 wt% Ca^2+^, SB1; 7.9 wt% salinity + 1.5 wt% Ca^2+^) and the D_H_-value remained almost unchanged ≤ 100 nm over the measured period of 30 days. The appearance in aggregation was observed (2–13%) when salinity increased to ≥ 12 wt%, known as the critical salt concentration, (SB2; 12 wt% salinity + 2.5 wt% Ca^2+^, SB3; 16 wt% salinity + 3.5 wt% Ca^2+^) only for ANPs fluid. However, DLS measurements gave the largest size distribution of particles > 100 nm for the particle suspensions (1000-cit-AQDs and 500-cit-ANPs) in synthetic brine SB4 (20 wt% salinity + 4.5 wt% Ca^2+^). Since the synthetic brines contains amounts of divalent ions, such as Ca^2+^, the critical salt concentration was lowered, which leaded to increase aggregation and sedimentation of particles at an earlier stage (Table 2). According to Cesarano’work^85^, polyelectrolyte polymers with rich available carboxylic acid sites per molecule, depending on pH value and ionic strength of solvent can be dissociated (i.e. COO^−^) or nondissociated (i.e. COOH). As the fraction dissociated increases from around 0 to 1, the polymer charge varies from neutral to highly negatively. Cesarano^85^ proved that increasing in the pH and salt concentration resulted in increasing of the dissociation and negative charge of the polymer. Since, the acid dissociation (pK_a_) of the acrylic acid groups is 4.6^86^ at pH 7, acrylamido-sulfonic acid/acrylic acid/amido polymer with carboxylic acid sites per molecule is adsorbed on alumina at pH 7. The COO^-^ groups on polymer are capable to form strong change transfer complexes with Al cations on the alumina surface that are not covered with citrates and enhance citrate ligands^87^. The assembly mechanism of the adsorption of a carboxylic acid at the alumina-water interface can be also described as a ligand exchange, where the hydroxyl group is exchanged for a carboxylate group^88^. According to the ligand exchange model, when a change of the surface charge can be happened if an additional carboxylate groups are present in the molecule that are not coordinated to the surface or an –OH_2_^+^ group which is the better leaving group is exchanged instead of a OH^−^ group^88^. Therefore, the additional COO^−^ groups on alumina surface via polymer coating both leading to a more negative particle charge, causing the carboxylic acid groups remaining in the fluid, resulting in an increase of the ionic strength and form bridge with Ca^2+^ in fluid to stable particles in harsh media. However, we could achieve a good resistant suspension includes two-step coating of particles. The one step, we functionalized them with citrate as a small ligand and continued by adsorption of polymer. Therefore, the formulated fluids of PE-citrate-coated-alumina in SWP (500-PE-cit-ANPsswp; 0.05 wt% of cit-ANPs powder + 0.1 wt% of PE in SWP, 500-PE-cit-AQDsswp; 0.05 wt% of cit-AQDs powder + 0.05 wt% of PE in SWP and 1000-PE-cit-AQDsswp; 0.1 wt% of cit-AQDs powder + 0.1 wt% of PE in SWP) was selected for EOR tests. These fluids had a good dispersion behavior in static states (explained in previous section) without any significant forming flocculation in brine at high temperature. In all tests, suspensions of alumina were termed as 500-PE-cit-ANPsswp (0.05 wt% of cit-ANPs powder + 0.1 wt% of PE in SWP), 500-PE-cit-AQDsswp (0.05 wt% of cit-AQDs powder + 0.05 wt% of PE in SWP) and 1000-PE-cit-AQDsswp (0.1 wt% of cit-AQDs powder + 0.1 wt% of PE in SWP).

**Table 1:** The derived experimental parameters for prepared nanofluids from PE-citrate-AQDs/ANPs nanostructures.

Particle ID	Nanoparticle concentration (cit-particles) (wt%)	Optimal polymer concentration in brines (wt%)					pH –value of nanofluids					Viscosity of nanofluids (mPa.s) at 25 °C				
		Basic fluid					Basic fluid					Basic fluid				
		SWP	SB1	SB2	SB3	SB4	SWP	SB1	SB2	SB3	SB4	SWP	SB1	SB2	SB3	SB4
PE-cit-AQDs	0.05	0.05	0.075	0.0875	0.10	0.125	7.1	7.1	7.1	7.1	7.1	1.010	1.016	1.025	1.034	1.054
PE-cit-AQDs	0.10	0.10	0.15	0.175	0.20	0.25	7.1	7.1	7.1	7.1	7.1	1.015	1.017	1.027	1.035	1.059
PE-cit-ANPs	0.05	0.10	0.15	0.175	0.20	0.25	7.1	7.1	7.1	7.1	7.1	1.018	1.017	1.028	1.035	1.058

**Table 2:**
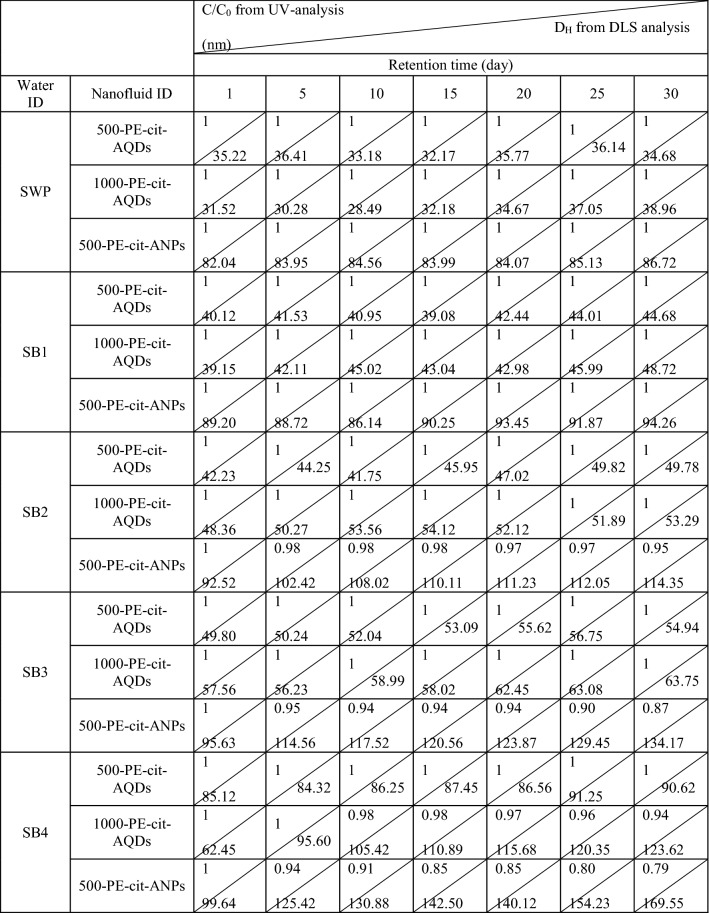
The salinity-dependent dispersion behavior of nanofluids at 90˚C from UV-analysis and DLS data.

### Wettability alteration

#### Contact angle measurements

**Figure 2:**
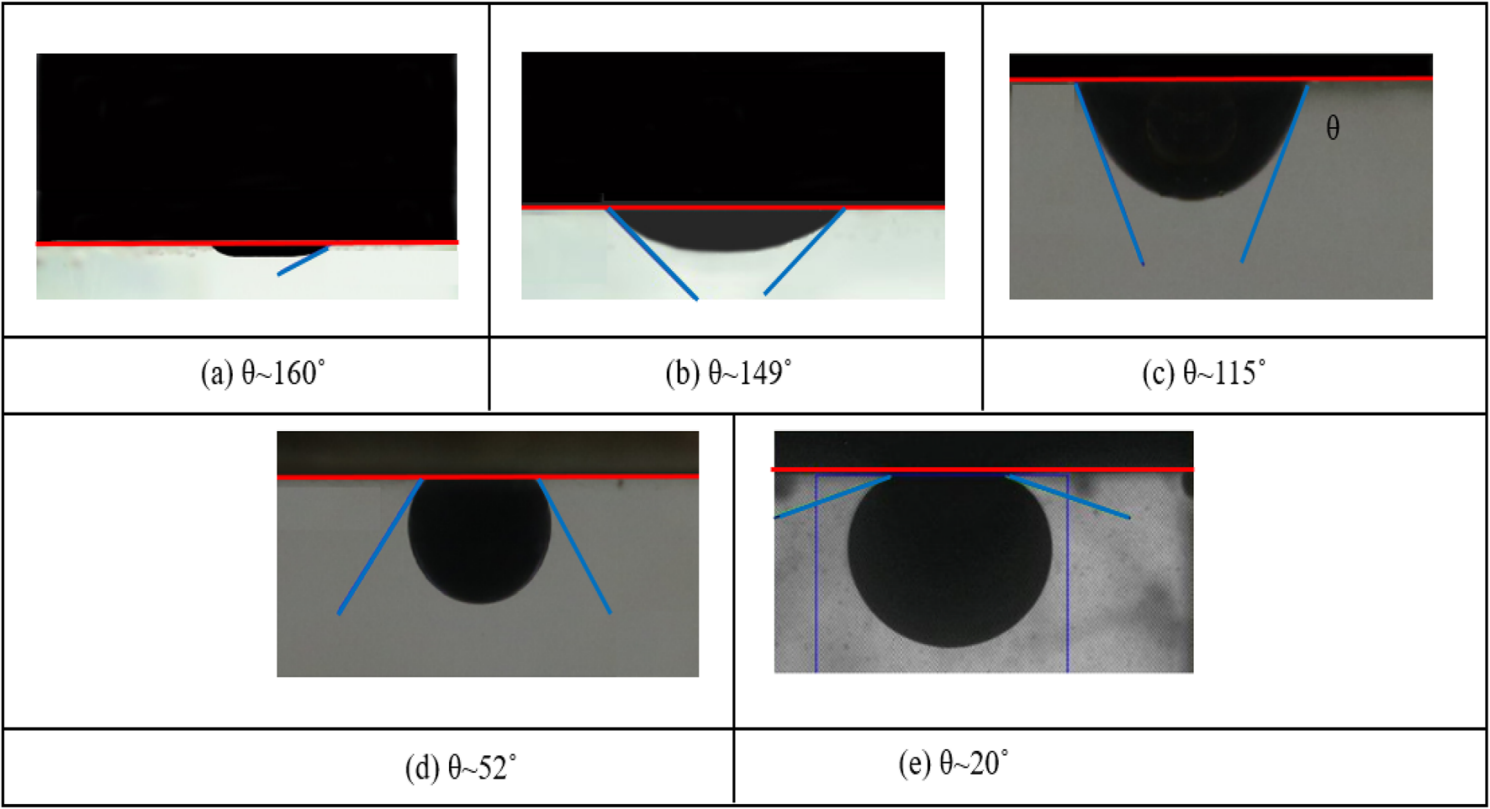
Contact angle measurements on the carbonate rock surface in solutions (**a**), reference oil-wet sample, (**b**) SWP without any particles, (**c**) 500-PE-cit-AQDs nanofluid, (**d**) 1000-PE-cit-AQDs nanofluid, (**e**) 500-PE-cit-ANPs nanofluid; Aging time = 72 h; Temperature = 90 °C, Pressure = 750 psi.

In order to assess the wettability alteration through contact angle measurement as a qualitative evaluation of wetting changes, several cleaned rock slices with 0.4 inches in diameter and 0.15 inches in thickness were used. The plates were aged for a week in crude oil at 90 °C in a pressurized vessel (750 psi) for oil-wet base. The initial oil-wet tendencies of them were measured, then submerged in a fluid having 500-PE-cit-ANPsswp, 500-PE-cit-AQDsswp and 1000-PE-cit-AQDsswp in a pressurized reactor at designed exposure aging time (72 h), temperature (90 °C) and pressure (750 psi). SWP was selected as a control test to compare any changes in angle. Sessile drop method was applied to measure contact angle for wettability alteration in ambient conditions. The results of these tests have been presented in Fig. 2. The results show that presence of Al_2_O_3_ in both quantum dots or nanoparticles had a crucial effect on the water-wet extent than SWP without any solids. When compared two samples with the same concentration of solids but different in size and morphology (500-PE-cit-AQDsswp and 500-PE-cit-ANPsswp), the surface wettability was more sensitive to the 500-PE-cit-ANPsswp suspension (~ 20°). The wettability tendency toward more water-wetness was also observed by increasing the solid extent in quantum suspensions (from ~ 115° for 500-PE-cit-AQDsswp to ~ 52° for 1000-PE-cit-AQDsswp). However, the contact angle measurements revealed that introducing the nanostructures both quantum and particle forms into the suspension resulted in wetting alteration from oil-wet to more water-wet condition and 500-PE-cit-ANPsswp and 1000-PE-cit-AQDsswp suspensions had the most enhancement.

#### Spontaneous imbibition in Amott cell

**Figure 3:**
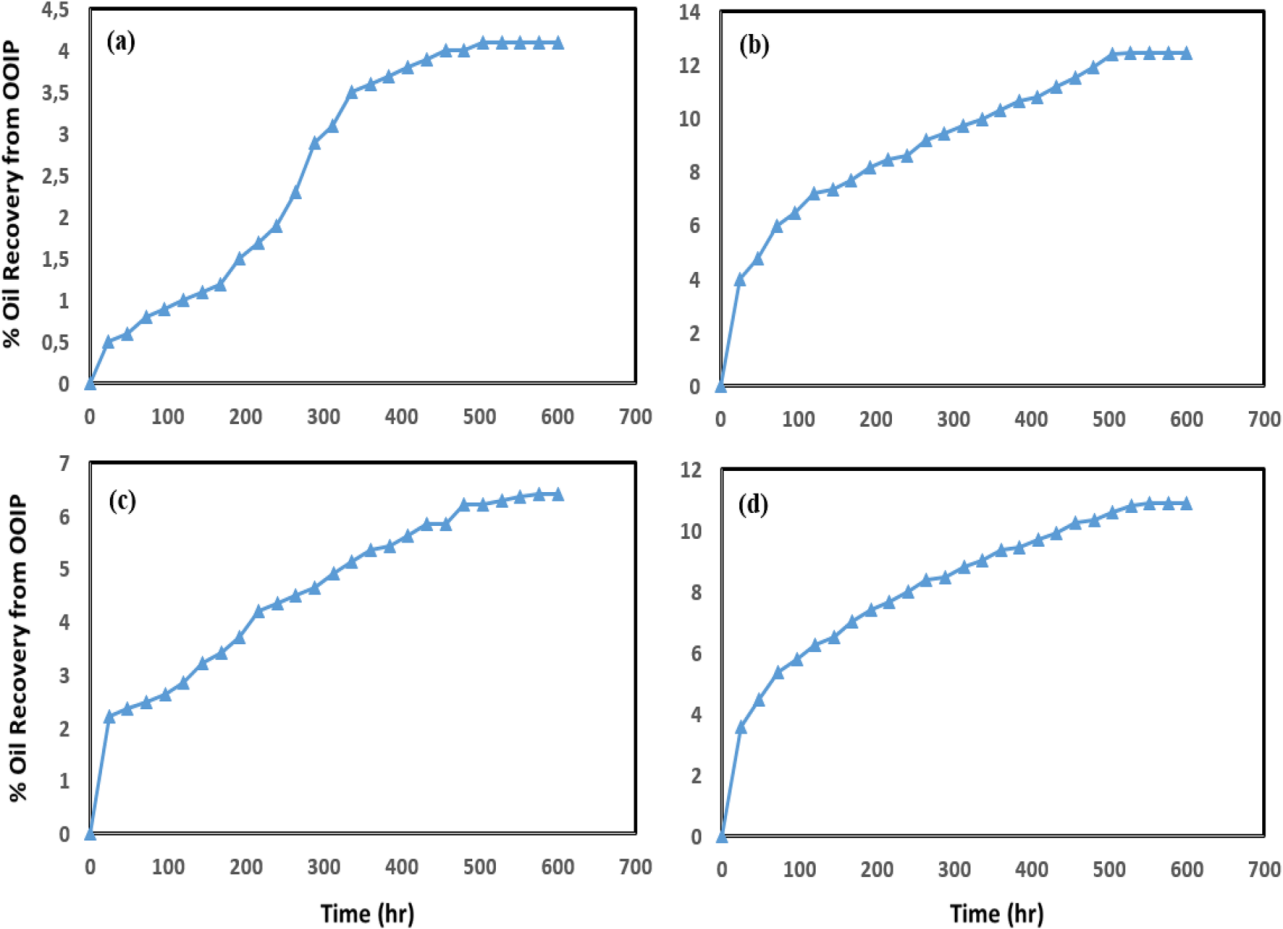
Spontaneous oil production from carbonate saturated-oil plugs in Amott cell at 90 °C (**a**) core II; treated with SWP (**b**) core A; treated with 500-PE-cit-ANPs nanofluid (**c**) core B; treated with 500-PE-cit-AQDs nanofluid (**d**) core C; treated with 1000-PE-cit-AQDs nanofluid.

The ultimate objective of spontaneous imbibition tests is to provide a quantitative assessment on the wettability alteration. In spite of the longevity of these tests, they are the most useful mechanism in recovery process from the hydrocarbon reservoirs, where the wetting fluid displaces the non-wetting fluid out of the pore-space and needed to confirm contact angle measurements^89^. In order to approach this objective, two sets of experiments were designed to give an enough understanding of spontaneous imbibition process. Four sister carbonate core plugs (A, B, C and II) were selected in the size of 3.8 cm in diameter and 6.3 cm in length (the initial rock properties have been shown in Table S2 supporting informatio). In the first step, the cleaned plugs were aged similar to the procedure mentioned in the previous section “PE-citrate-alumina complex” (the initially oil-saturated plugs at 90 °C for 40 days to provide oil-wet surfaces), then, the planned experiments were performed on them. Initially, the oil-wet-saturated plugs A (OOIP = 9.19 cc), B (OOIP = 7.95 cc), C (OOIP = 9.42 cc) and II (OOIP = 11.21 cc) were placed in a core-flood apparatus and treated with fluid injection to alter the wetting property from oil-wet to an intermediate state. Core samples were injected with SWP and three various of nanofluids (500-PE-cit-ANPsswp, 500-PE-cit-AQDsswp and 1000-PE-cit-AQDsswp). Core-flood displacement experiments were done to investigate the behavior of the injected fluid in the presence of crude oil on four cores at reservoir conditions (at 90 °C; backpressure of 750 psi). We performed three runs, SWP or nanofluid flooding until no oil produced (Run#1), a particular rest time for fluid effect (72 h) (Run#2) and finally SWP injection until no oil produced (Run#3). In all injection runs, a constant flow rate of 0.2 cc/min was satisfied. In each flooding run, the volume of oil produced was measured carefully and the remained oil in place was calculated. Finally, we saturated treated-samples with crude oil again at room temperature. The difference between the initial and final weight of cores corresponds to the amount of oil in place. In the second step, the re-oil-saturated cores A (treated with 500-PE-cit-ANPsswp), B (treated with 500-PE-cit-AQDsswp), C (treated with 1000-PE-cit-AQDsswp) and II (treated with SWP) were soaked in Amott cells contain in SWP as well^83^. The Amott cells were then brought to reservoir temperature and amount of spontaneously produced oil was recorded versus time for each cell until no more oil was recovered. In this study, SWP-treated core II was selected as the base case and any variations of the parameters were compared to it. Figure 3a–d compares the behavior of imbibition of SWP into core plugs. The total amount of spontaneous oil production was obtained 4.2% from OOIP for SWP-treated plug II during 25 days (see Fig. 3a). As it is evident from these Figs. 3b–d, SWP, however, imbibed more into the core plugs, resulted in an improvement in the amount of spontaneous produced oil from 6.42 to 12.42% for particle-treated samples during 25 days. These results show that the nano suspensions injected into core plugs had a positive impact on wetting and could alter rock surface wettability to more water wet, which could enhance spontaneous imbibition. A minimum produced oil of 6.42% in core sample B (treated with 500-PE-cit-AQDsswp) and a maximum produced oil of 12.42% in core sample A (treated with 500-PE-cit-ANPsswp) was observed. Moreover, adding of cit-AQDs up to 0.1 wt% to the injected suspension into core C, caused in more water-wet tendency followed more spontaneous imbibition of SWP in it. Figure 3 represents that the results for cores A (treated with 500-PE-cit-ANPsswp) and C (treated with 1000-AQDsswp) were more encouraging than that of core B (treated with 500-PE-cit-AQDsswp). Comparing the sets of experiments show that the ultimate recovery owing to core A (treated with 500-PE-cit-ANPsswp) was about 8.38% higher than the recovery obtained by core II (base case) and these values reached to 2.22% and 6.29% for cores B (treated with 500-PE-cit-AQDsswp) and C (treated with 1000-PE-cit-AQDsswp) higher than it, respectively, which was in accordance with measured contact angle data. However, the more response of the system to the imbibition of SWP into the particle-treated cores than base case could be related to the wettability alteration due to nanofluids. Spontaneous imbibition tests showed we could assure that wettability alteration be the well-accepted mechanism in oil recovery in nanofluid-based EOR experiments. Since, nanofluids at these concentrations ≤ 0.1 wt% was not sensitive to interfacial tension (IFT), it seems that particle-flooding into oil-wet plug samples, separates the remaining oil adhering to the rock surface by wettability alteration to more water-wetness or disjoining pressure mechanism^90^. One of the important issues based on these results was that, the presence of Al_2_O_3_ in crystalline form even with lower concentration could alter the carbonate surface charges toward most water-wet condition.

#### Core-flooding test scenarios (brine/nanofluid/brine injection)

**Figure 4:**
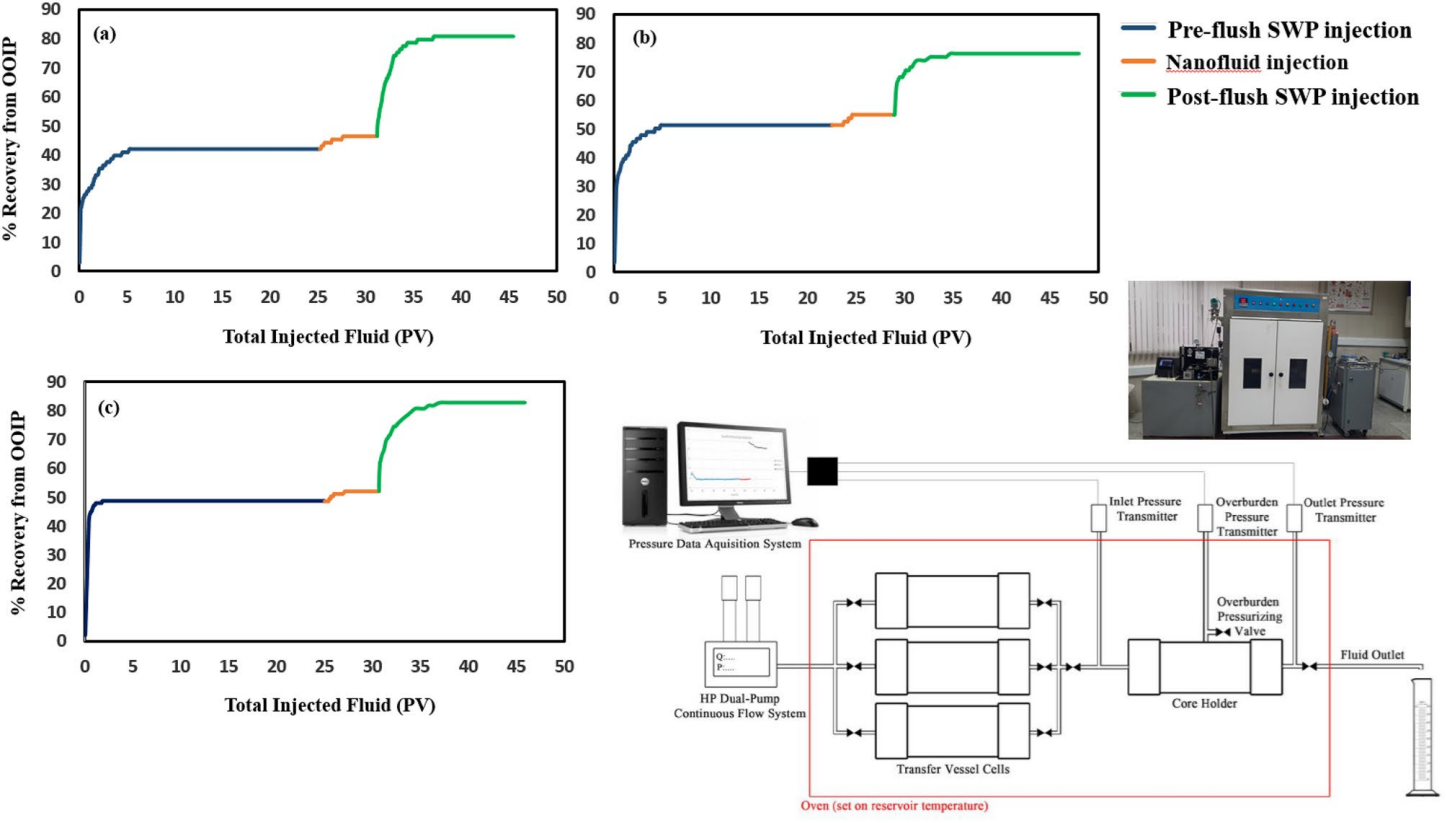
Oil recovery performance vs. injected PV of the core plugs at 90 °C, 750 psi and retention time of 72 h (**a**) core D, SWP/500-PE-cit-ANPs nanofluid/retention time/SWP injection scenario (**b**) core E, SWP/500-PE-cit-AQDs nanofluid/retention time/SWP injection scenario (**c**) core F, SWP/1000-PE-cit-AQDs nanofluid/retention time/SWP injection scenario.

**Figure 5:**
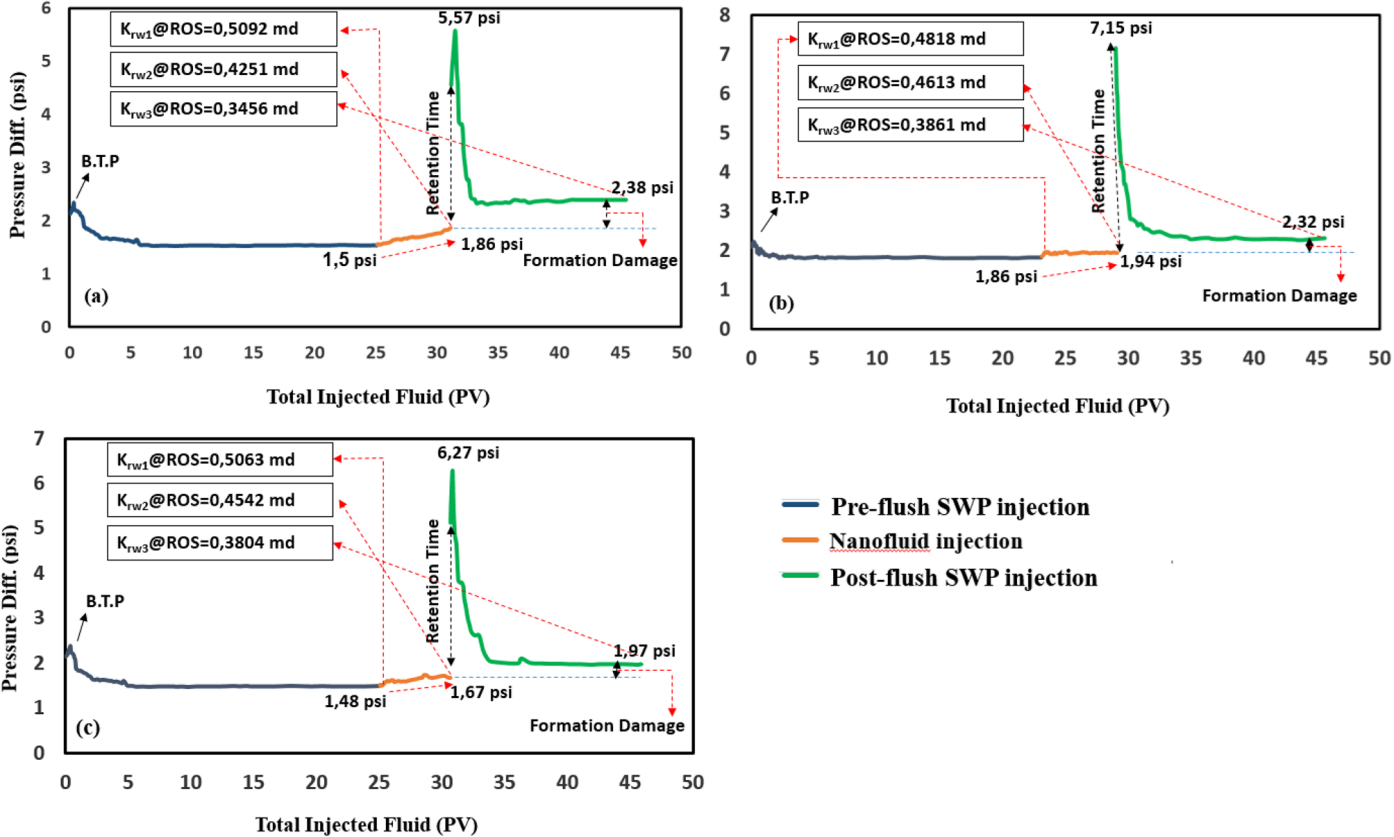
Differential pressure profile during pre-flush water flooding, nano flooding and post-flush water flooding again across the core plugs at 90 °C and 750 psi and retention time of 72 h (**a**) core D, SWP/500-PE-cit-ANPs nanofluid/retention time/SWP injection scenario (**b**) core E, SWP/500-PE-cit-AQDs nanofluid/retention time/SWP injection scenario (**c**) core F, SWP/1000-PE-cit-AQDs nanofluid/retention time/SWP injection scenario.

The purpose of these experiments was to compare the residual oil reduction with and without any particles. Core-flood displacement experiments investigated the behavior of the nanofluids in the presence of crude oil on three oil-wet-saturated core plugs with an absolute permeability of 16.5 md (core D, 500-PE-cit-ANPsswp), 15.3 md (core E, 500-PE-cit-AQDsswp) and 18.16 md (core F, 1000-PE-cit-AQDsswp) in comparison to that with SWP at reservoir pressure and temperature (at 90 °C, backpressure of 750 psi). The initially oil-saturated plugs were done at 90 °C for 40 days to provide oil-wet surfaces. The initial rock properties have been shown in (Table S2 supporting information). The objective of these tests was to study the wetting properties of nanofluids on oil recovery at reservoir conditions. These phenomena were plotted in Fig. 4. The experiments were run in scenario of SWP/nanofluid/retention time/SWP injection at reservoir conditions (backpressure of 750 psi and 90 °C). In all injection runs, a constant flow rate of 0.2 cc/min was satisfied. From Fig. 4, at first, water-flooding was conducted approximately 25 PVs until no oil produced. The pre-flush brine-flooding, oil recovery was achieved 42.24%, 51.41%, and 48.68% of original oil in place (OOIP) for cores D, E and F, respectively. Due to the insensitivity of nanofluids to reducing interfacial tension, during nanofluid injection no significant oil was recorded. Displacement efficiency during nanofluid injection was obtained very low (approx., 2–3%). However, we observed that displacement efficiency was higher when retention time was increased up to 72 h. After this time, with post-flush water flooding, the residual oil saturation decreased significantly. The reduction of residual oil saturation was obtained about 38.88%, 24.94% and 34.19% for cores D, E and F, respectively. Comparison oil recovery in the second brine-flooding with initial water injection showed additional oil recovery of about 24–38% due to nanofluid effect after retention time would be significant and the core-flooding results corroborated the efficiency of nanofluid in enhanced oil recovery. Also, as observed in Fig. 4a and c, the enhanced oil recovery due to a less concentration state by 500-PE-cit-ANPs was consistent with 1000-PE-cit-AQDs at higher concentrations. The differential pressure during the core plugs was also registered by precision Siemens DP gauge with range 0–30 bar. Figure 5a shows relationship between pressure drop and injected pore volumes. At pre-flush water injection, 2-phase flow existed and differential pressure across the plug D was increased from 2.13 to in a maximum value of 2.34 psi at breakthrough point while dropped to a value of 1.5 psi and stabilized at this point. There was no more oil produced after 6 PVs of brine-flooding and the injection was stopped at 24 PVs. During nanofluid injection (500-PE-cit-ANPs) for about 2 PVs, the differential pressure increased significant from 1.5 to 1.86 psi. After the retention time, the brine-flooding as post-flush was started again. It was observed that the differential pressure across the core increased quickly to a maximum value of about 5.57 psi at the beginning of this stage and then dropped down to 2.38 psi during injection and stabilized at this point. However, there was no more oil produced after 17 PVs of injection brine-flooding in third section and the injection was stopped at 19 PVs. This phenomenon was repeated for cores E and F. Breakthrough was observed in 2.23 and 2.38 psi for cores E and F, respectively. However, in all tests, the pressure drop during nanofluid injection and post-flush brine period were just slightly higher than pre-flush brine injection (0.88 psi for core D, 0.46 psi for core E and 0.49 psi for core F) (see Fig. 5). After whole injection process, plug’s permeability (k_rw3_ @ROS) was reduced to 0.345, 0.386 and 0.380 md for cores D, E and F, respectively at the end of post-flush brine injection that has been impaired dramatically to make core almost impermeable. This reason may be due to aggregation and/or irreversible adsorption of nanoparticles to block the pore throats and the formation of mineral deposits due to incompatibility of SWP and formation water.

#### Formation damage due to mineral deposition and/or alumina release

Pragmatic implementation of water or nanofluids at reservoir conditions, requires thorough and significant focusing on the scale formation that associated mainly with the following reasons:

- Mineral deposition due to incompatibility between fluids, especially in water injection cases and reservoir fluids which leads to the scale formation if they interact chemically when mixed.
- Adsorption (reversible or irreversible) and retention of alumina nanoparticles in the porous media.

The ultimate purpose of this section is to investigate both the potential of different formation damages containing mineral scale and/or alumina release through permeability-reduction measurement at reservoir conditions. Two approaches were used in formation damage experiments. First, investigation of incompatibility between water injection without any particles and reservoir fluids (cores D’, E’ and F’, these plug samples were the same cores D, E and F that restored by enforcing cleaning procedures). Second, consideration of adsorption/detachment and straining behavior of nanoparticles in porous media (second step): (cores D”, E” and F”, these rocks were the same cores D’, E’ and F’ that restored by applying cleaning procedures again). Experimental works were performed on three long carbonate core plugs at each run which were categorized into single-phase core flood experiments in absence of oil. To ensure test constancy and result reliability, three similar water-wet carbonate cores was selected. Due to restrictions on core plug supply, we restored the rock samples of D, E and F (used in core-flood experiments) by implementing cleaning procedures. Rock samples were cleaned through the soxhlet apparatus in toluene and methanol sequentially during 2 weeks, then followed by drying at 100 °C overnight and vacuum to remove all air from them.

#### Dynamic fluid–fluid incompatibility tests

In general, scale formation in water flooding often results from incompatibility between sea water, which included a great amount of sulfate ions (SO_4_^–2^) and formation water with strongly binding of cation ions (Ca^+2^, Ba^+2^, Sr^+2^) or due to a change in temperature, pressure and the pH-value when water flows from one location to another^91^. When two incompatible waters contact each other, sulfate ions in the composition of sea water causes the formation of solid scales (CaSO_4_, BaSO_4_, SrSO_4_) upon mixing with the cation-rich formation water, causes significant permeability decline^92,93^. Also, reduction in the solubility of the salts can lead to precipitation of carbonate scales such as CaCO_3_^94^. In this section, we mainly evaluated permeability reduction of porous media due to the scale-formation tendency of SWP without any particles in contact with FW at reservoir conditions. With regard to the water-analysis data (Table S1 supporting information), SWP had a total salinity of 41,780 ppm and contained significant amounts of SO_4_^2^. Formation water was used for rock saturation and an absolute liquid permeability determined (Table S1 supporting information). Core-flooding scenarios of SWP/retention-time/SWP injection was employed for cleaned water-wet cores D’, E’ and F’ (at 90 °C; backpressure of 750 psi). Before any run, the cores were reached 100% saturation with FW by using a vacuum pump at room temperature and absolute permeability determined. Initially, 9 PVs of SWP was flooded into FW-saturated core and mixed with FW inside the porous media until no differential pressure across the core changed. Afterwards, when retention time was increased up to 72 h, SWP was injected as a post-flush to investigate the effect of formation damage due to incompatible waters. In all injection runs, a constant flow rate of 0.2 cc/min, temperature of 90 °C and backpressure of 750 psi was satisfied. Differential pressure across the core samples was recorded continuously during the whole injection process. The end of each run, the permeability of core was calculated with Darcy’s equation to observe the effect of mineral scale on permeability decline. Finally, the core samples were removed, cleaned with methanol through soxhlet extraction apparatus at 70˚C and dried in an oven at 100 °C for a day.

#### Dynamic nanofluids transport experiments

The strain behavior of nanostructures was studied in SWP at reservoir conditions. The same sets of experiments were performed for the suspensions having different sizes and percentages of particles (500-PE-cit-AQDs, 1000-PE-cit-AQDs and 500-PE-cit-ANPs). Core-flooding scenarios of nanofluid/retention-time/SWP injection was applied for three core samples D”, E” and F” at 90 °C. Formation water was first injected into the water-wet core plugs and continued long enough to ensure 100% saturation and absolute permeability determined. Then, about of 9 PVs of nanofluids as dispersing phase were injected into the FW-saturated core plugs to investigate the effect of nanoparticle adsorption and retention on permeability reduction. Residence time was kept 72 h. Afterwards; a tertiary injection process was applied to flush retained nanoparticles by SWP injection. Therefore, 9 PVs of SWP was flooded as post-flush to observe the reversible/irreversible adsorption of particles. The resulting permeability due to nanofluid injection was also calculated with Darcy’s equation.

#### Interpretation of permeability decline

We defined average percentage liquid permeability impairment due to mineral scale and total average percentage liquid permeability impairment due to both mineral and alumina retention at end-point of both flooding and rest time in as follows:

3 $${\mathrm{K}}^{\mathrm{^{\prime}}}\mathrm{or }{\mathrm{K}}^{"}=\frac{({\mathrm{k}}_{\mathrm{abs}}-{\mathrm{k}}_{\mathrm{i}}) }{{\mathrm{k}}_{\mathrm{abs}}}\times 100,\mathrm{ i}=\mathrm{1,2},3$$

where, k_abs_ is absolute liquid permeability (md), k_i_ is permeability (md), K’ is related to percentage liquid permeability impairment due to mineral deposition in brine/brine injection scenario and K” is associated with percentage formation damage due to the total of mineral scale and alumina retention in nanofluid/brine flooding scenario.

The difference between the total formation damage (K”_i_) and impairment due to mineral scale (K’_i_) corresponds to the amount of damage creates by alumina particles as follows:

4 $${\mathrm{\% P}.\mathrm{I}.\mathrm{A}=\mathrm{K}}_{\mathrm{i}=\mathrm{1,2},3}^{"} (\mathrm{nanofluid }/\text{brine} \, \text{scenario })-{\mathrm{K}}_{\mathrm{i}=\mathrm{1,2},3}^{\mathrm{^{\prime}}} (\mathrm{brine }/\text{brine} \, \text{scenario })$$

**Figure 6:**
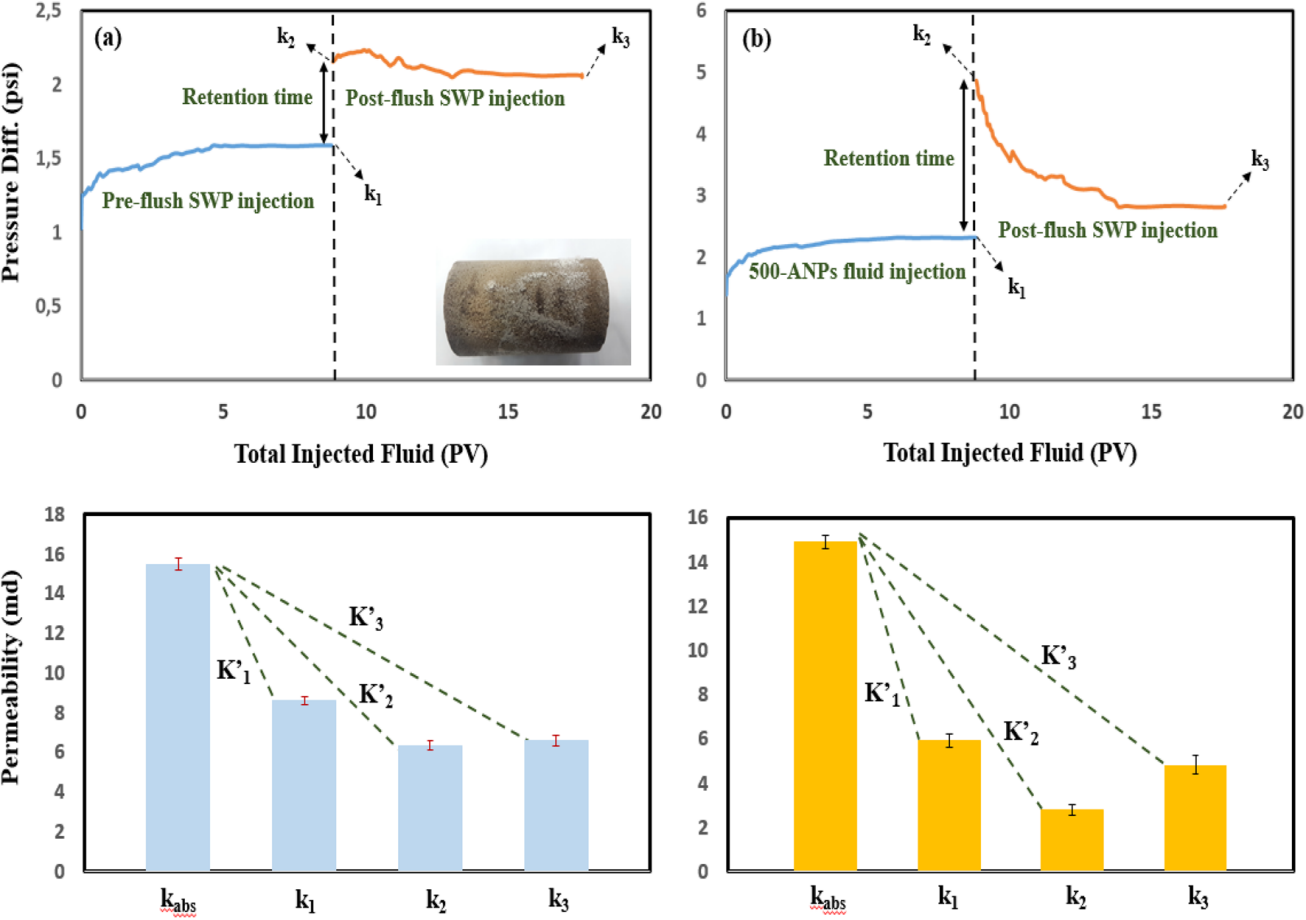
Differential pressure curves for fluid injection experiment in absence of oil for (**a**) core D’, SWP/retention time/SWP (**b**) core D”, 500-PE-cit-ANPs nanofluid/retention time/SWP, retention time = 72 h.

**Figure 7:**
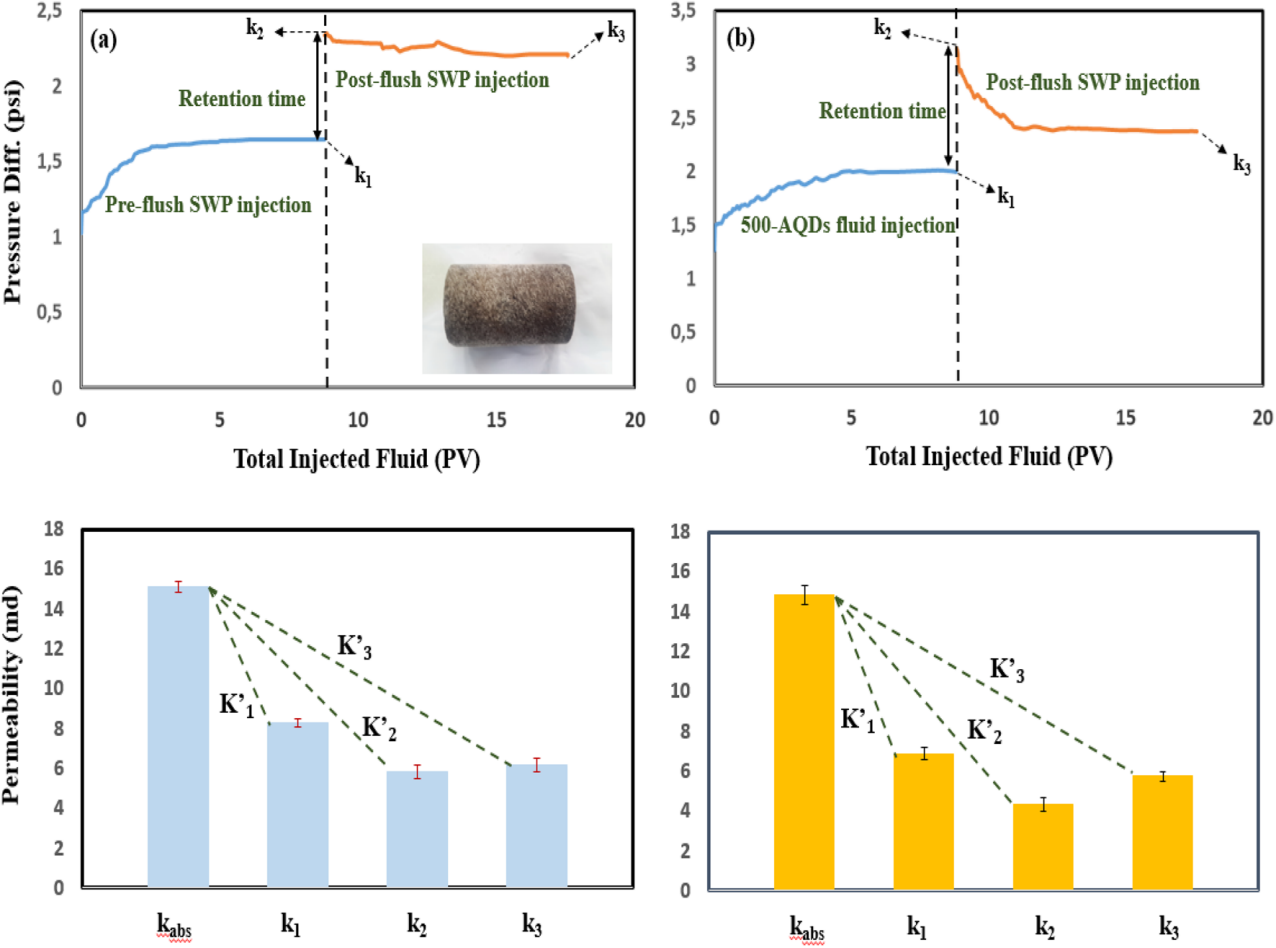
Differential pressure curves for fluid injection experiment in absence of oil for (**a**) core E’, SWP/retention time/SWP (**b**) core E”, 500-PE-cit-AQDs nanofluid/retention time/SWP, retention time = 72 h.

**Figure 8:**
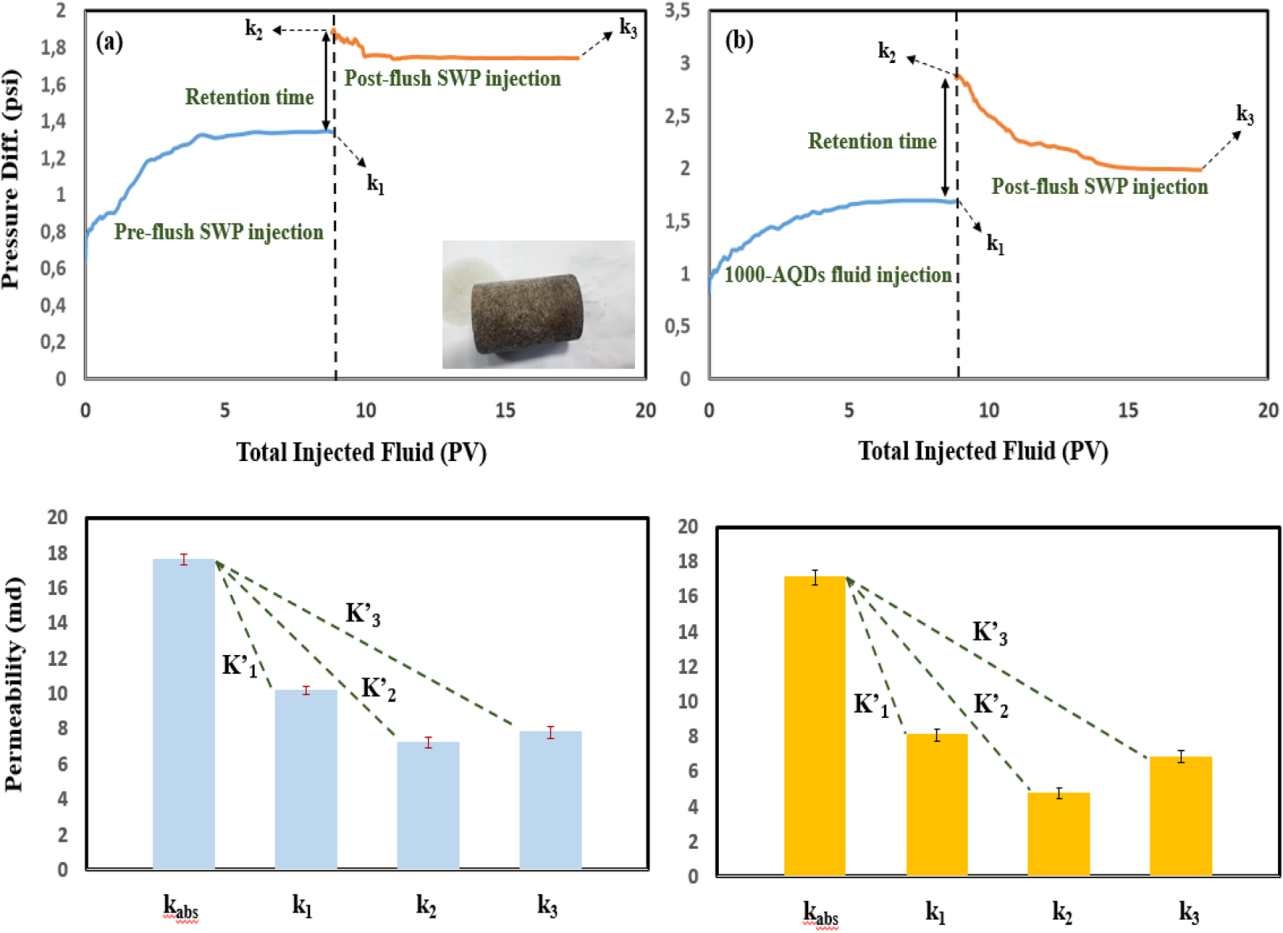
Differential pressure curves for fluid injection experiment in absence of oil for (**a**) core F’, SWP/retention time/SWP (**b**) core F”, 1000-PE-cit-AQDs nanofluid/retention time/SWP, retention time = 72 h.

Figures 6, 7, and 8a and b compares differential pressure across the core plugs during fluid (SWP and nanofluids) transport experiments. All the related data for the permeability impairment calculations have been presented in Table 3. i.e. From Fig. 6a, the differential pressure increased up from 1.25 psi to a maximum value of 1.58 psi during SWP injection (~ 9 PV) and stabilized at this point. After a particular time (72 h), it was observed that the ΔP climbed quickly to a maximum point of 2.15 psi at the beginning of the post-flush SWP into the rock and then slightly lowered to an end-point value of 2.06 psi. Extend of permeability loss caused by mineral scale was observed clearly in the rock pores varied in different situations. About 44.32% initial permeability loss was obtained in pre-flush (Table 3; K’_1_; core D’) but more than loss in permeability (59.07%) occurred at rest time (Table 3; K’_2_; core D’), finally; reached to a value of 57.29% in post-flush flooding (Table 3; K’_3_, core D’). Comparing the findings from the scale formation study (K’_2_ and K’_3_) indicated that mineral blocking the pore throats could not be flushed out of core significantly by post-flush flooding (approx. 2%; Table 3). Figure 6b shows transport behavior of SWP-based nanofluid (500-PE-cit-ANPs) into the rock (core D”). 500-ANPs nanofluid exhibited a higher different pressure behavior than core D’. The differential pressure increased during pre-flush and stabilized at a value of 2.31 psi, resulting in a percentage decline in permeability of 60.19% (K”_1_; core D”; Fig. 6b). Moreover, Bigger permeability decline changes were revealed during retention time and post-flush SWP transport (81.15%, K”_2_ and 67.52%, K”_3_; core D”). The post-flush injection declined the impairment approximately 13.63% (K”_2_ minus K”_3_, core D”). Nevertheless, adsorbed nanoparticles could not be flushed out of core fully. At post-SWP flooding as shown in Fig. 6b, the permeability impairment was higher than in post-SWP flush as shown in Fig. 6a which was interpreted some nanoparticles had blocked the pore networks. Comparing the relevant data from the impairment study in Table 3 (K”_3core D”_ and K’_3core D’_) indicates that 57.29% of blocking the pores was related to mineral deposition and remaining (10.23%) attributed to alumina irreversible adsorption. Also, the core samples E”, F” yielded lower differential pressure curves when flooded with 0.05 wt% and 0.1wt% of AQDs. As seen from the ΔP recording in Figs. 7b and 8b, the pressure started increasing immediately when the quantum-based fluids came in contact with the cores (E”, F”). However, the differential pressure was lower (less than 5–20%) than injecting nanoparticle-based fluids (see Figs. 6b, 7b and 8b). With regard to the data in Table 3, there was a big jump in permeability improvement in post-flush injection into the rocks (E” and F”). Comparing the findings from the total scale formation during rest time (Table 3; K”_2_), (mineral deposition + particle release) inside core plugs E” and F” with core samples E’ and F’ (having only mineral damage, Table 3; K’_2_), when a quantum-based nanofluid was used as an injection, the permeability impairment of core samples injected with nanofluids was sensible, with a minimum impairment of 9.42% in core sample E” and a maximum impairment of 13.26% in core sample F”. An improvement up to 77% of the post-flush injection was seen for the most successful core floods E” and F” in Table 3, which means that the permeability impairment with quantum-based nanofluids even bigger concentration was not severe. However, at residence time, incompatibility phenomena reduced permeability significantly in all core plugs up to 61% (core D’, K’_2_, 59.07%; core E’, K’_2_, 61.30%; core F’, K’_2_, 58.81%). Additionally, during the initial flow period and residence time, cores D”, E” and F”, resulted in a larger permeability reduction than in cores D’, E’ and F’, despite being flooded under seemingly similar conditions that could be attributed to the particle impairment. Mineral blocking the pore throats could not be removed out of cores significantly but more than 35–73% of adsorbed alumina could remove from cores D”, E” and F” during the post-flush period. γ-Al_2_O_3_ reduced permeability up to 10.23% even with lower concentration, when flooded into porous media. No significant impairment (2–4%) was observed for quantum-based nanofluids. Figure 8b shows that the addition of AQDs up to 0.1 wt% had little effect on both the pressure increase and the permeability impairment. In general, when nanoparticles pass thorough pore throats, adsorption, desorption, blocking, transport and aggregation of nanoparticles can occur. Blocking will take place if the diameter of the particles is larger than pore neck. Aggregation of nanoparticles happens if the previous equilibrium system breaks up and nanoparticles form clusters^44^. When the total force of five forces such as attractive van der Waals forces, double layer repulsion forces, born repulsion forces, acid–base interactions and hydrodynamics is negative, the attraction between nanoparticles and pore walls is larger than repulsion between nanoparticles, which leads to adsorption of them on the pore throats^95^. Otherwise desorption of nanoparticles from the pore walls will occur. Adsorption and desorption is a dynamic balance process controlled by the total force between nanoparticles and pore walls^96^. Adsorption and transport behavior of nanoparticles inside porous media play a very critical role for wettability alteration^96,97^. Zhang et al.^96^ described that both reversible and irreversible adsorption of nanoparticles can occur during injection into porous media. Li et al.^98^ investigated the impact of nanoparticle adsorption on transport and wettability alteration in water-wet Bera sandstone. Their results showed that different types of nanoparticles have different adsorption and desorption behavior and different ability to impair the permeability. Also, Arian et al. explained reversible and irreversible adsorption of bare and hybrid silica nanoparticles onto carbonate surface at reservoir condition^99^. According to Al-Anssari’work, nanoparticles are practiced both reversible and irreversible adsorption on carbonate surface^100^. As pointed out by wasan et al^90^, the wettability of rock–oil–water systems can be altered by nanoparticles when adsorbs on surface solid that creates a wedge-film structure in the three-phase contact area, wetting wedge of a drop or bubble on a solid surface happens near the three-phase contact, which promotes the spreading of liquids containing nanoparticles and makes the system more water-wet^90^. In this study, the effect of nanostructures (AQDs & ANPs) on wettability alteration was investigated by measuring residual oil production due to spontaneous imbibition in Amott cell and the contact angle. These typical tests maybe could prove that the mechanism leading to improved oil recovery in carbonate rock due to these particles might be associated to wettability alteration. However, because of both impairment and improvement phenomena in permeability reduction tests together wettability alteration measurements might be confirmed adsorption (reversible or irreversible) behavior of alumina both in quantum or nanoparticle structures.

**Table 3:** Permeability reduction of core plug samples during fluid flooding and impairment behavior due to mineral deposition and nanoparticles.

Injection scenarios	Core ID	k_abs_ (md)	k_1_ (md)	k_2_ (md)	k_3_ (md)	K’_1_	K’_2_	K’_3_	K”_1_	K”_2_	K”_3_	Impairment due to mineral deposition (%)	Impairment due to alumina particles (%)
Brine/brine	D’	15.508	8.636	6.346	6.624	44.32	59.07	57.29	–	–	–	57.29	–
500-PE-cit-ANPs/brine	D”	14.903	5.931	2.807	4.838	–	–	–	60.19	81.15	67.52	–	10.23
Brine/brine	E’	15.122	8.291	5.850	6.188	45.16	61.30	59.07	–	–	–	59.07	–
500-PE-cit-AQDs/brine	E”	14.846	6.882	4.345	5.851	–	–	–	53.84	70.85	61.42	–	2.35
Brine/brine	F’	17.651	10.182	7.269	7.803	42.31	58.81	55.79	–	–	–	55.79	–
1000-PE-cit-AQDs/brine	F”	17.107	8.127	4.777	6.861	–	–	–	52.47	72.07	59.88	–	4.09

### Comparison between EOR performance of AQDs & ANPs

The wettability alteration through contact angle measurements, spontaneous imbibition tests and adsorption and retention of nanoparticles experiments by various concentrations and types of nanostructures revealed that there were different behaviors between AQDs & ANPs nanofluids. When nanostructures were at the same concentration (500-PE-cit-AQDsswp & 500-PE-cit-ANPsswp), ANPs could change wettability deeply to more water-wet conditions than AQDs (Fig. 2c,e). While at concentration higher than 0.05 wt% for AQDs (1000-PE-cit-AQDs), experimental results showed that 500-PE-cit-ANPs could change wettability slightly to more water-wet than 1000-PE-cit-AQDs again (Fig. 2d,e). The reason might be related to fundamental differences include structure, surface energy and size-value between AQDs & ANPs particles. As previously explained, the samples investigated in this study appear to be synthesized in-lab and supplied commercial, so the surface energy of pure them is not a well-defined quantity in this study. ANPs exist as bigger particles and crystalline shape have more strong adsorption and retention inside core than AQDs. Because the basic mechanism for wettability alteration due to nanoparticles is adsorption and retention of them inside core, therefore, ANPs could change wettability significantly at lower concentration than AQDs. Also, laboratory results of nanoparticles transport showed that AQDs & ANPs had different adsorption and retention behavior inside core. When 500-PE-cit-ANPs was injected into the core, nanofluid exhibited a higher differential pressure behavior than 1000-PE-cit-AQDs nanofluid (Figs. 6b, 8b). Also, based on the data in Table 3, only ~ 35% of adsorbed ANPs could flush out of core, while, increased up to 70% for AQDs particles. It may be explained that the adsorption of ANPs accrues on pore walls according to multilayer. In other hands, ANPs with larger particles and crystalline shape can grow on the pore walls and make adsorption layer thicker and thicker until blockage of pore channels. Tavakoli et al.^101^ proved that the lower surface energy of amorphous alumina compared with crystalline type makes this phase the most energetically stable phase. Thus, thereby, ANPs with crystalline shape having the higher surface energy, easily aggregate to bigger particles with hard agglomeration, as a result, the adsorption of ANPs on pore walls will be multilayer. Adsorption of ANPs is almost strong and most of adsorption is irreversible. While the adsorption of AQDs in amorphous type on pore walls may be close to monolayer due to smaller in size, lower surface energy and less pressure drop. This indicate that adsorption of AQDs doesn’t reduce pore channel throat size or most of AQDs adsorption is reversible. These results are partially in agreement with those found in Li’ work^102^. Li et al. investigated the impact of adsorption and transport of two types of silica nanoparticles (hydrophilic silica nano-structure and hydrophilic silica colloidal nanoparticles) on wettability alteration of intermediate wet Bera sandstone. Pressure drop curves showed that adsorption and retention of nano-structure particles inside core was significant while colloidal nanoparticles did not adsorb much. Their results showed that the nanoparticles undergo both adsorption and desorption as well as retention during injection. Based on our findings in this study, 500-PE-cit-ANPs nanofluid had better adsorption ability than 1000-PE-cit-AQDs nanofluid, but adsorption 500-PE-cit-ANPs could block pore channels resulting in more permeability reduction, while adsorption of 1000-PE-cit-AQDs didn’t impair permeability significantly.

## Conclusions

In the present work, the potential of using two types of coated-alumina particles (PE-cit-AQDs and PE-cit-ANPs) was investigated as a mean to enhance traditional EOR techniques in order to further increase oil and less reservoir impairment. The following conclusions were made from the analyzing observations and the results of tests:

- Long-time stability of particles was examined in SWP and elevated temperature (90 °C) for up to 1 month. Colloidal dispersion of alumina particles was achieved with citrate ligand and a sufficient level of carboxylate-sulfonate-based polyelectrolyte polymer. The optimal amount of alumina particles in aqueous suspension was obtained 0.05 wt% for ANPs, increased up to 0.1 wt% for AQDs.
- The wettability alteration results and spontaneous imbibition tests upon exposure to particles showed that both 1000-PE-cit-AQDs and 500-PE-cit-ANPs nanofluids were significant effective than 500-PE-cit-AQDs nanofluid to alter wettability of oil-wet carbonate surface to more water-wet condition.
- The quantum-based suspensions, as a new formulated nanofluid, enhanced oil recovery from reservoir rock at higher concentrations (0.1 wt%). A considerable increase in oil production was observed for 500-PE-cit-ANPs and 1000-PE-cit-AQDs nanofluids (34–38%) from OOIP through core-flood displacement experiments.
- Permeability-impairment measurements revealed that ~ 55–57% of blocking the pores was related to mineral deposition.
- It was found that quantum-based Al_2_O_3_ nanofluids had the lowest impairment (~ 2–4%) and γ-alumina-based nanofluids had higher impairment (~ 10%) on the carbonate grains’ surfaces. “

## Methodology

### Materials

Aluminum nitrate nonahydrate, hexamethylenetetramine, citric acid, potassium nitrate, potassium hydroxide, calcium chloride dehydrate and sodium chloride were provided by Merck company. In this work, γ-Al_2_O_3_ nanoparticles commercial powder (< 99.99%, provided by the Scharlau company) and α-Al_2_O_3_ quantum dots (synthesized in-house according to Nemade’work^62^) were used as alumina nanoparticles (ANPs) and quantum dots (AQDs)-based nanofluids. A carboxylate-sulfonate-based polyelectrolyte polymer (acrylamido-sulfonic acid/acrylic acid/ acryl amido) was designed for stabilization of alumina particles at high reservoir conditions.

### Crude oil

Light aromatic crude oil with 40˚ American Petroleum Institute (API) gravity and around 0.2% content of asphaltene, which made it a proper candidate for experimental EOR tests, was provided from an Iranian carbonate reservoir. The oil was filtered through a 0.5 µm filter to remove any solid contaminates.

### Brines

The sea water (Persian Gulf sea water) with a pH-value of 7.1 was used as ionic strength media both as the main base dispersant fluid and as the injected water in all EOR experiments. The different synthetic brines were also prepared to investigate the effects of high salinity and cation-rich media (Ca^2+^) on stability of nanofluids. They were prepared using pure chemicals such as sodium chloride, calcium chloride and deionized water with a conductivity of 1.34 × 10^–4^ s/m. Formation water, FW (from an Iranian carbonate reservoir) was used for rock saturation and incompatibility tests. The ionic compositions of these waters have been included in (Table S1 supporting information). For convenience, SWP was Persian Gulf sea water; SB1, SB2, SB3 and SB4 were synthetic brines.

### Laboratory EOR experiments

EOR tests were studied to investigate the role of size, particle morphology and concentration effect of alumina particles with enough focus over the trend of different parameters changes underground reservoir conditions. Both the contact angle measurements and the Amott cell tests were used to evaluate the qualitative and quantitative order of wettability changes of the rock surfaces, including carbonate slices and core plugs. Dynamic core displacements were conducted to test the water-nanofluid-oil flow and formation damage behaviors in porous media at reservoir conditions. To prepare oil-wet samples, the cleaned core plugs (collected from a carbonate reservoir in Iran) were saturated with formation water in vacuum system under pressure for 24 h, then flooded with it to calculate liquid absolute permeability, finally, flooded again with the crude oil to reach irreducible water saturation (S_wi_) and placed in a pressurized crude containing vessel (750 psi) at 90 °C for 40 days. These rock samples were carbonate, which were typically dolomite. The physical properties and description of core samples have been listed in (Table S2 supporting information).

## Supplementary Information

Supplementary Information.

## Data Availability

All importance data generated or analyzed during this study are included in this published article [and its supplementary information files] and a limited number of others, if needed available from the corresponding author on reasonable request.

## Abbreviations

ANPs: Alumina nanoparticles
AQDs: Alumina quantum dots
ASAP: Accelerated surface area and porosimetry
C/C_0_: Relative concentration of remained nanostructures in suspension
DLS: Dynamic light scattering
k: Permeability (md)
NP: Nanoparticles
OOIP: Original oil in place
PE: Polyelectrolyte
