# Supplementary material for: Newly engineered alumina quantum dot-based nanofluid in enhanced oil recovery at reservoir conditions
Source: Sci Rep. 2022 Jun 9;12:9505. doi: 10.1038/s41598-022-12387-y (PMC9184488; doi:10.1038/s41598-022-12387-y)
Supplement: Supplementary file 1 — Supplementary Information. [file 41598_2022_12387_MOESM1_ESM.pdf]

Newly engineered alumina quantum dot-based nanofluid in Enhanced oil recovery at Reservoir Conditions

Nosrat Izadi[1], Bahram Nasernejad[1*]

[1]Faculty of Chemical Engineering, Amirkabir University of Technology, Hafez Ave, P.O.Box 15875-4413, Tehran, Iran.

*Corresponding Author at: Chemical Engineering Department, Amirkabir University of Technology (Tehran Polytechnic), P.O.Box 15875-4413, Iran

*Corresponding Author Email: banana@aut.ac.ir

Supporting Information

1. Characterizations and analysis method

The phase identification and crystalline structure of nanoparticles was determined by X-ray Diffraction. XRD pattern of samples was recorded on a PW-1840 X-ray diffract meter (Philips) using Cu-K_α (1.5 A°) radiation operated at 40 kV and 30 mA. The crystalline phases were identified with the help of the JCPDS cards. The surface morphology of nanostructures was obtained using Transmission Electron Microscopy (TEM). TEM photographs were obtained with a LEO-912-AB operated at 85 Kv. Accelerated surface area and porosimetry (ASAP) analysis was performed to determine the specific surface area and total pore volume of nanostructures. ASAP was carried out with sub critical nitrogen at 77.3 K on a Micromeritics American Instrument (model 2010), which is calibrated by using a surface area reference material Al_2O_3. An infrared spectrometer was used to obtain Fourier transform infrared spectroscopy of citrate coated of particles. FT-IR spectra was recorded on Perkin Elmer Spectrum-GX. A fluorescence measurement was made for synthesized alumina quantum dots from 300 to 500 nm by PL analyzer. PL analysis were measured by Spectrometer Spectroscopy, Avaspec 2048 TEC. Size distribution of nanoparticles was measured using dynamic light scattering analyzer (Malvern

Instrument Inc, London, UK). Sedimentation experiments were conducted with a UV-visible spectrometer at a wavelength of 520 nm.

2. Properties of injected fluids

Properties of injected fluids have been shown in Table S4.

Table S4. Properties of injected fluids in this study

	FW*	SWP**	Crude oil	500-PE-cit-ANPs-fluid	500-PE-cit-AQDs-fluid	1000-PE-cit-AQDs-fluid
Density (g/cm3) at 25˚C	1.18	-	-	-	-	-
Viscosity (cP) at 25˚C	1.3	-	0.85	-	-	-
Viscosity (cP) at 90˚C	-	0.493	-	0.495	0.493	0.494

*FW: formation water

**SWP: Gulf Persian sea water

3. Coreflood apparatus

The setup includes: (1) a high pressure dual-pump continuous flow system, (2) three transfer vessel cells, (3) a Hassler type core holder, (4) a backpressure system, (5) an oven to maintain the reservoir temperature and (6) digital pressure data acquisition system. (Figure 4).

4. Core flooding procedure

In this section, we fully described how to prepare one core plug sample (i.e core D in Table S2) for core displacement experiments.

4.1. Step 1: Calculation of PV (pore volume)

The core plug was selected from a carbonate reservoir and had a length of 6.4 cm and a diameter of 3.8 cm (Table S2). The cleaned core sample was initially 100% saturated with formation water (FW) in the vacuum set up under pressure for 24 hours at room temperature. The difference between the initial and final weight of core corresponds to the amount of water that saturated the pore throats. This amount of water divided to density of FW (1.18 g/cm^3 at 25˚C; Table 4S) corresponds to pore volume of core (PV=13.50 cc).

4.2. Step 2: Calculation of absolute liquid permeability

The saturated core plug from step 1 was flooded with FW to calculate absolute liquid permeability. Darcy's equation (Eq. 1) was used to in the interpretation of permeability. Table S5 shows the experimental permeability measurement data.

$$k = \frac{Q \times \mu \times L}{A \times \Delta P} \times 1000 \qquad (1)$$

Where:

k: permeability (md)

Q: volumetric flow rate of liquid (cc/s);

L: length of core (cm); L=6.4 cm

A: cross section of core (cm^2); A=11.3 cm^2

μ: viscosity of FW at 25˚C (cP); μ =1.3 cp

ΔP: differential pressure across the core (bar)

Table S5. Experimental permeability measurement data

Q (cc/min)	ΔP (psi)
0.2	2.15
0.4	4.33
0.6	6.57
0.8	8.72

The slope of the curve changes of ((Q.μ/A)×1000) via ΔP/L corresponds to permeability of core (k=16.501 md).

4.3. Step 3: Calculation of S_{wi} and OOIP

The core plug was then flooded with the crude oil at room temperature until irreducible water saturation was reached. Irreducible water saturation and original oil in place was calculated using Eq 2 and 3. (S_{wi}% =25.16; OOIP=10.11 cc; Table S2)

$$S_{wi}(\%) = \frac{PV - v_{pw}}{PV} \times 100 \qquad (2)$$

$$OOIP = PV - v_{rw} \qquad (3)$$

Where:

V_{pw}: volume of produced water from the core (cc)

V_{rw}: volume of remained water inside the core (cc)

PV: pore volume of the core (cc)

4.4. Step 4: Aging of core

Finally, the core sample was placed in a pressurized vessel containing crude oil at 750 psi and 90 °C for 21 days. This aging procedure could change the rocks wettability from initial water-wet state to relatively oil-wet state.

4.5. Step 5: Coreflooding experiments at reservoir conditions

The flooding procedure for core sample had three stages: pre-flush injected water: 25 PVs into the aged core plug from step 4 with irreducible water saturation (S_{wi}) at injection rate of 0.2 cc/min, until the water cut reached 99% and no more oil produced. Nanofluid injection: 6 PVs at the same rate as tertiary recovery mode, Post-flush injected water: 14 PVs again until reaching second residual oil saturation. The pressure difference between two faces of the core plug and the volume of recovered fluids were recorded continuously along the test. Table S6 shows the total experimental data in this step.

Breakthrough point was happened at PV=0.2 cc fore core D. These phenomena were plotted in Figure 4a. Figure 5a shows relationship between pressure drop and injected pore volumes.

Table S6. Total experimental data in coreflooding scenarios for core D											
				Volumes after being corrected for Tot. dead Vol. =11.73 (except for third step)			Volume separately for each part injection		Cumulative Volumes		
Injected fluid	Total injected vol (cc)	Water produced (cc)	Oil produced (cc)	Total injected vol (cc)	Water produced (cc)	Oil produced (cc)	Total injected vol (PV)	Oil produced from OOIP %	Total injected vol (PV)	Oil produced from OOIP %	Differential pressure (psia)
Pre-flush water injection											
water	0,5	0,0	0,50	-11,23	0,0	-11,23	-	-	-	-	-
water	1	0,0	1,00	-10,73	0,0	-10,73	-	-	-	-	-
water	1,5	0,0	1,50	-10,23	0,0	-10,23	-	-	-	-	-
water	2	0,0	2,00	-9,73	0,0	-9,73	-	-	-	-	-
water	2,6	0,0	2,60	-9,13	0,0	-9,13	-	-	-	-	-
water	3	0,0	3,00	-8,73	0,0	-8,73	-	-	-	-	-
water	3,5	0,0	3,50	-8,23	0,0	-8,23	-	-	-	-	-
water	4	0,0	4,00	-7,73	0,0	-7,73	-	-	-	-	-
water	4,2	0,0	4,20	-7,53	0,0	-7,53	-	-	-	-	-
water	4,5	0,0	4,50	-7,23	0,0	-7,23	-	-	-	-	-
water	4,8	0,0	4,80	-6,93	0,0	-6,93	-	-	-	-	-
water	5	0,0	5,00	-6,73	0,0	-6,73	-	-	-	-	-
water	5,2	0,0	5,20	-6,53	0,0	-6,53	-	-	-	-	-
water	5,4	0,0	5,40	-6,33	0,0	-6,33	-	-	-	-	-
water	5,6	0,0	5,60	-6,13	0,0	-6,13	-	-	-	-	-

water	5,8	0,0	5,80	-5,93	0,0	-5,93	-	-	-	-	-
water	6	0,0	6,00	-5,73	0,0	-5,73	-	-	-	-	-
water	6,3	0,0	6,30	-5,43	0,0	-5,43	-	-	-	-	-
water	6,8	0,0	6,80	-4,93	0,0	-4,93	-	-	-	-	-
water	7,1	0,0	7,10	-4,63	0,0	-4,63	-	-	-	-	-
water	7,5	0,0	7,50	-4,23	0,0	-4,23	-	-	-	-	-
water	7,8	0,0	7,80	-3,93	0,0	-3,93	-	-	-	-	-
water	8,1	0,0	8,10	-3,63	0,0	-3,63	-	-	-	-	-
water	8,5	0,0	8,50	-3,23	0,0	-3,23	-	-	-	-	-
water	8,9	0,0	8,90	-2,83	0,0	-2,83	-	-	-	-	-
water	9,2	0,0	9,20	-2,53	0,0	-2,53	-	-	-	-	-
water	9,5	0,0	9,50	-2,23	0,0	-2,23	-	-	-	-	-
water	9,8	0,0	9,80	-1,93	0,0	-1,93	-	-	-	-	-
water	10,1	0,0	10,10	-1,63	0,0	-1,63	-	-	-	-	-
water	10,3	0,0	10,30	-1,43	0,0	-1,43	-	-	-	-	-
water	10,7	0,0	10,70	-1,03	0,0	-1,03	-	-	-	-	-
water	11	0,0	11,00	-0,73	0,0	-0,73	-	-	-	-	-
water	11,5	0,0	11,50	-0,23	0,0	-0,23	-	-	-	-	-
water	12,0	0,0	12,00	0,27	0,0	0,27	0,02	2,67	0,02	2,67	2,18

water	12,2	0,0	12,20	0,47	0,0	0,47	0,03	4,65	0,03	4,65	2,22
water	12,5	0,0	12,50	0,77	0,0	0,77	0,06	7,62	0,06	7,62	2,24
water	12,8	0,0	12,80	1,07	0,0	1,07	0,08	10,58	0,08	10,58	2,27
water	13,0	0,0	13,00	1,27	0,0	1,27	0,09	12,56	0,09	12,56	2,29
water	13,7	0,0	13,70	1,97	0,0	1,97	0,15	19,49	0,15	19,49	2,31
water	13,9	0,0	13,90	2,17	0,0	2,17	0,16	21,46	0,16	21,46	2,33
water	14,4	0,5	13,90	2,67	0,5	2,17	0,20	21,46	0,20	21,46	2,34
water	15,1	1,1	14,00	3,37	1,1	2,27	0,25	22,45	0,25	22,45	2,23
water	15,3	1,3	14,00	3,57	1,3	2,27	0,26	22,45	0,26	22,45	2,21
water	15,5	1,5	14,00	3,77	1,5	2,27	0,28	22,45	0,28	22,45	2,21
water	15,9	1,7	14,20	4,17	1,7	2,47	0,31	24,43	0,31	24,43	2,20
water	16,2	2,0	14,20	4,47	2,0	2,47	0,33	24,43	0,33	24,43	2,19
water	16,5	2,3	14,20	4,77	2,3	2,47	0,35	24,43	0,35	24,43	2,19
water	17,0	2,7	14,30	5,27	2,7	2,57	0,39	25,42	0,39	25,42	2,17
water	17,7	3,4	14,30	5,97	3,4	2,57	0,44	25,42	0,44	25,42	2,16
water	18,2	4,0	14,20	6,47	4,0	2,47	0,48	24,43	0,48	24,43	2,11
water	19,3	4,9	14,40	7,57	4,9	2,67	0,56	26,41	0,56	26,41	2,09
water	20,3	5,9	14,40	8,57	5,9	2,67	0,63	26,41	0,63	26,41	2,08
water	21,4	7,0	14,40	9,67	7,0	2,67	0,72	26,41	0,72	26,41	2,00

water	22,5	8,0	14,50	10,77	8,0	2,77	0,80	27,40	0,80	27,40	1,89
water	23,6	9,1	14,50	11,87	9,1	2,77	0,88	27,40	0,88	27,40	1,88
water	24,5	10,0	14,50	12,77	10,0	2,77	0,95	27,40	0,95	27,40	1,86
water	25,5	10,9	14,60	13,77	10,9	2,87	1,02	28,39	1,02	28,39	1,86
water	26,6	11,9	14,70	14,87	11,9	2,97	1,10	29,38	1,10	29,38	1,84
water	27,6	13,0	14,60	15,87	13,0	2,87	1,18	28,39	1,18	28,39	1,86
water	28,8	14,2	14,60	17,07	14,2	2,87	1,26	28,39	1,26	28,39	1,84
water	29,7	14,9	14,80	17,97	14,9	3,07	1,33	30,37	1,33	30,37	1,83
water	30,7	16,0	14,70	18,97	16,0	2,97	1,41	29,38	1,41	29,38	1,83
water	31,2	16,5	14,70	19,47	16,5	2,97	1,44	29,38	1,44	29,38	1,81
water	31,4	16,6	14,80	19,67	16,6	3,07	1,46	30,37	1,46	30,37	1,80
water	32,3	17,4	14,90	20,57	17,4	3,17	1,52	31,36	1,52	31,36	1,80
water	32,8	17,8	15,00	21,07	17,8	3,27	1,56	32,34	1,56	32,34	1,79
water	33,9	19,0	14,90	22,17	19,0	3,17	1,64	31,36	1,64	31,36	1,78
water	34,9	19,8	15,10	23,17	19,8	3,37	1,72	33,33	1,72	33,33	1,75
water	35,9	20,8	15,10	24,17	20,8	3,37	1,79	33,33	1,79	33,33	1,74
water	37,1	22,1	15,00	25,37	22,1	3,27	1,88	32,34	1,88	32,34	1,74
water	38,0	22,9	15,10	26,27	22,9	3,37	1,95	33,33	1,95	33,33	1,73
water	39,0	23,8	15,20	27,27	23,8	3,47	2,02	34,32	2,02	34,32	1,71

water	40,1	24,8	15,30	28,37	24,8	3,57	2,10	35,31	2,10	35,31	1,68
water	41,2	25,9	15,30	29,47	25,9	3,57	2,18	35,31	2,18	35,31	1,67
water	42,3	27,0	15,30	30,57	27,0	3,57	2,26	35,31	2,26	35,31	1,67
water	43,2	27,9	15,30	31,47	27,9	3,57	2,33	35,31	2,33	35,31	1,67
water	44,2	28,9	15,30	32,47	28,9	3,57	2,41	35,31	2,41	35,31	1,68
water	45,2	29,8	15,40	33,47	29,8	3,67	2,48	36,30	2,48	36,30	1,66
water	46,2	30,9	15,30	34,47	30,9	3,57	2,55	35,31	2,55	35,31	1,65
water	48,4	33,0	15,40	36,67	33,0	3,67	2,72	36,30	2,72	36,30	1,66
water	50,4	34,9	15,50	38,67	34,9	3,77	2,86	37,29	2,86	37,29	1,66
water	52,5	37,0	15,50	40,77	37,0	3,77	3,02	37,29	3,02	37,29	1,66
water	54,5	39,1	15,40	42,77	39,1	3,67	3,17	36,30	3,17	36,30	1,66
water	56,7	41,0	15,70	44,97	41,0	3,97	3,33	39,27	3,33	39,27	1,65
water	58,7	43,1	15,60	46,97	43,1	3,87	3,48	38,28	3,48	38,28	1,64
water	60,8	45,1	15,70	49,07	45,1	3,97	3,63	39,27	3,63	39,27	1,64
water	62,4	46,6	15,80	50,67	46,6	4,07	3,75	40,26	3,75	40,26	1,63
water	63,9	48,2	15,70	52,17	48,2	3,97	3,86	39,27	3,86	39,27	1,63
water	66,0	50,3	15,70	54,27	50,3	3,97	4,02	39,27	4,02	39,27	1,63
water	68,1	52,4	15,70	56,37	52,4	3,97	4,18	39,27	4,18	39,27	1,62
water	70,4	54,6	15,80	58,67	54,6	4,07	4,35	40,26	4,35	40,26	1,61

water	72,5	56,6	15,90	60,77	56,6	4,17	4,50	41,25	4,50	41,25	1,60
water	73,8	58,0	15,80	62,07	58,7	4,07	4,60	40,26	4,60	40,26	1,64
water	76,4	60,6	15,80	64,67	60,6	4,07	4,79	40,26	4,79	40,26	1,55
water	78,5	62,6	15,90	66,77	62,6	4,17	4,95	41,25	4,95	41,25	1,54
water	80,6	64,7	15,90	68,87	64,7	4,17	5,10	41,25	5,10	41,25	1,54
water	82,6	66,7	15,90	70,87	66,7	4,17	5,25	41,25	5,25	41,25	1,53
water	85,7	69,8	15,90	73,97	69,8	4,17	5,48	41,25	5,48	41,25	1,53
water	88,9	72,9	16,00	77,17	72,9	4,27	5,72	42,24	5,72	42,24	1,53
water	93,0	77,1	15,90	81,27	77,1	4,17	6,02	41,25	6,02	41,25	1,53
water	98,2	82,2	16,00	86,47	82,2	4,27	6,41	42,24	6,41	42,24	1,53
water	103,4	87,4	16,00	91,67	87,4	4,27	6,79	42,24	6,79	42,24	1,53
water	115,9	99,9	16,00	104,17	99,9	4,27	7,72	42,24	7,72	42,24	1,53
water	128,9	112,9	16,00	117,17	112,4	4,27	8,68	42,24	8,68	42,24	1,53
water	143,9	127,9	16,00	132,17	127,9	4,27	9,79	42,24	9,79	42,24	1,52
water	155,3	139,3	16,00	143,57	139,4	4,27	10,63	42,24	10,63	42,24	1,53
water	166,8	150,8	16,00	155,07	150,8	4,27	11,49	42,24	11,49	42,24	1,53
water	181,3	165,3	16,00	169,57	165,3	4,27	12,56	42,24	12,56	42,24	1,53
water	193,8	177,8	16,00	182,07	177,8	4,27	13,49	42,24	13,49	42,24	1,53
water	207,3	191,3	16,00	195,57	191,3	4,27	14,49	42,24	14,49	42,24	1,54

water	215,6	199,6	16,00	203,87	199,6	4,27	15,10	42,24	15,10	42,24	1,54
water	222,8	206,8	16,00	211,07	206,9	4,27	15,63	42,24	15,63	42,24	1,53
water	233,2	217,2	16,00	221,47	217,3	4,27	16,41	42,24	16,41	42,24	1,54
water	243,6	227,6	16,00	231,87	227,6	4,27	17,18	42,24	17,18	42,24	1,53
water	255,6	239,6	16,00	243,87	239,6	4,27	18,06	42,24	18,06	42,24	1,53
water	264,4	248,4	16,00	252,67	248,6	4,27	18,72	42,24	18,72	42,24	1,53
water	271,7	255,7	16,00	259,97	255,7	4,27	19,26	42,24	19,26	42,24	1,54
water	280,0	264,0	16,00	268,27	264,0	4,27	19,87	42,24	19,87	42,24	1,54
water	284,1	268,1	16,00	272,37	268,2	4,27	20,18	42,24	20,18	42,24	1,54
water	290,4	274,4	16,00	278,67	274,4	4,27	20,64	42,24	20,64	42,24	1,53
water	295,5	279,5	16,00	283,77	279,6	4,27	21,02	42,24	21,02	42,24	1,54
water	302,8	286,8	16,00	291,07	286,6	4,27	21,56	42,24	21,56	42,24	1,53
water	311,1	295,1	16,00	299,37	295,2	4,27	22,18	42,24	22,18	42,24	1,53
water	325,7	309,7	16,00	313,97	309,7	4,27	23,26	42,24	23,26	42,24	1,53
water	338,1	322,1	16,00	326,37	322,2	4,27	24,18	42,24	24,18	42,24	1,53
water	350,6	334,6	16,00	338,87	334,6	4,27	25,10	42,24	25,10	42,24	1,53
water	350,6	334,6	16,00	338,87	334,6	4,27	25,10	42,24	25,10	42,24	1,54
water	350,6	334,6	16,00	338,87	334,6	4,27	25,10	42,24	25,10	42,24	1,54
water	350,6	334,6	16,00	338,87	334,6	4,27	25,10	42,24	25,10	42,24	1,53

water	350,6	334,6	16,00	338,87	334,6	4,27	25,10	42,24	25,10	42,24	1,54
water	350,6	334,6	16,00	338,87	334,6	4,27	25,10	42,24	25,10	42,24	1,54
water	350,6	334,6	16,00	338,87	334,6	4,27	25,10	42,24	25,10	42,24	1,55
water	350,6	334,6	16,00	338,87	334,6	4,27	25,10	42,24	25,10	42,24	1,55
	Nanofluid injection (500-ANPs)										
nanofluid	0,5	0,5	0,00	-11,23	-11,23	0,00	-	-	-	-	-
nanofluid	1,0	1,0	0,00	-10,73	-10,73	0,00	-	-	-	-	-
nanofluid	1,5	1,5	0,00	-10,23	-10,23	0,00	-	-	-	-	-
nanofluid	2,0	2,0	0,00	-9,73	-9,73	0,00	-	-	-	-	-
nanofluid	2,5	2,5	0,00	-9,23	-9,23	0,00	-	-	-	-	-
nanofluid	3,0	3,0	0,00	-8,73	-8,73	0,00	-	-	-	-	-
nanofluid	3,5	3,5	0,00	-8,23	-8,23	0,00	-	-	-	-	-
nanofluid	4,0	4,0	0,00	-7,73	-7,73	0,00	-	-	-	-	-
nanofluid	4,6	4,6	0,00	-7,13	-7,13	0,00	-	-	-	-	-
nanofluid	5,0	5,0	0,00	-6,73	-6,73	0,00	-	-	-	-	-
nanofluid	5,5	5,5	0,00	-6,23	-6,23	0,00	-	-	-	-	-
nanofluid	6,0	6,0	0,00	-5,73	-5,73	0,00	-	-	-	-	-
nanofluid	6,6	6,6	0,00	-5,13	-5,13	0,00	-	-	-	-	-
nanofluid	7,0	7,0	0,00	-4,73	-4,73	0,00	-	-	-	-	-
nanofluid	7,5	7,5	0,00	-4,23	-4,23	0,00	-	-	-	-	-

nanofluid	8,0	8,0	0,00	-3,73	-3,73	0,00	-	-	-	-	-
nanofluid	8,4	8,4	0,00	-3,33	-3,33	0,00	-	-	-	-	-
nanofluid	9,0	9,0	0,00	-2,73	-2,73	0,00	-	-	-	-	-
nanofluid	9,6	9,6	0,00	-2,13	-2,13	0,00	-	-	-	-	-
nanofluid	10,1	10,1	0,00	-1,63	-1,63	0,00	-	-	-	-	-
nanofluid	10,8	10,8	0,00	-0,93	-0,93	0,00	-	-	-	-	-
nanofluid	11,5	11,5	0,00	-0,23	-0,23	0,00	-	-	-	-	-
nanofluid	12,6	12,6	0,00	0,87	0,87	0,00	0,06	0,00	25,16	42,24	1,56
nanofluid	13,6	13,6	0,00	1,87	1,87	0,00	0,14	0,00	25,24	42,24	1,57
nanofluid	15,1	15,1	0,00	3,37	3,37	0,00	0,25	0,00	25,35	42,24	1,56
nanofluid	16,2	16,2	0,00	4,47	4,47	0,00	0,33	0,00	25,43	42,24	1,56
nanofluid	17,2	17,1	0,10	5,47	5,36	0,11	0,41	1,09	25,51	43,33	1,57
nanofluid	19,3	19,2	0,10	7,57	7,46	0,11	0,56	1,09	25,66	43,33	1,58
nanofluid	22,4	22,3	0,10	10,67	10,56	0,11	0,79	1,09	25,89	43,33	1,58
nanofluid	25,6	25,4	0,20	13,87	13,65	0,22	1,03	2,18	26,13	44,42	1,59
nanofluid	28,6	28,4	0,20	16,87	16,65	0,22	1,25	2,18	26,35	44,42	1,62
nanofluid	29,7	29,5	0,20	17,97	17,75	0,22	1,33	2,18	26,43	44,42	1,64
nanofluid	32,8	32,6	0,20	21,07	20,85	0,22	1,56	2,18	26,66	44,42	1,65
nanofluid	34,9	34,6	0,30	23,17	22,83	0,34	1,72	3,36	26,82	45,60	1,66

nanofluid	37,0	36,7	0,30	25,27	24,93	0,34	1,87	3,36	26,97	45,60	1,66
nanofluid	38,9	38,6	0,30	27,17	26,83	0,34	2,01	3,36	27,11	45,60	1,64
nanofluid	41,1	40,8	0,30	29,37	29,03	0,34	2,18	3,36	27,28	45,60	1,66
nanofluid	43,0	42,7	0,30	31,27	30,93	0,34	2,32	3,36	27,42	45,60	1,67
nanofluid	45,3	45,0	0,30	33,57	33,23	0,34	2,49	3,36	27,59	45,60	1,67
nanofluid	46,3	46,0	0,30	34,57	34,23	0,34	2,56	3,36	27,66	45,60	1,68
nanofluid	48,4	48,0	0,40	36,67	36,22	0,45	2,72	4,45	27,82	46,69	1,69
nanofluid	50,5	50,1	0,40	38,77	38,32	0,45	2,87	4,45	27,97	46,69	1,69
nanofluid	51,5	51,1	0,40	39,77	39,32	0,45	2,95	4,45	28,05	46,69	1,70
nanofluid	53,6	53,2	0,40	41,87	41,42	0,45	3,10	4,45	28,20	46,69	1,69
nanofluid	54,8	54,4	0,40	43,07	42,62	0,45	3,19	4,45	28,29	46,69	1,69
nanofluid	56,1	55,7	0,40	44,37	43,92	0,45	3,29	4,45	28,39	46,69	1,69
nanofluid	57,7	57,3	0,40	45,97	45,52	0,45	3,41	4,45	28,51	46,69	1,69
nanofluid	59,3	58,9	0,40	47,57	47,12	0,45	3,52	4,45	28,62	46,69	1,70
nanofluid	61,9	61,5	0,40	50,17	49,72	0,45	3,72	4,45	28,82	46,69	1,70
nanofluid	64,0	63,6	0,40	52,27	51,82	0,45	3,87	4,45	28,97	46,69	1,71
nanofluid	65,0	64,6	0,40	53,27	52,82	0,45	3,95	4,45	29,05	46,69	1,71
nanofluid	66,8	66,4	0,40	55,07	54,62	0,45	4,08	4,45	29,18	46,69	1,72
nanofluid	68,1	67,7	0,40	56,37	55,92	0,45	4,18	4,45	29,28	46,69	1,73

nanofluid	70,3	69,9	0,40	58,57	58,12	0,45	4,34	4,45	29,44	46,69	1,73
nanofluid	72,3	71,9	0,40	60,57	60,12	0,45	4,49	4,45	29,59	46,69	1,74
nanofluid	74,6	74,2	0,40	62,87	62,42	0,45	4,66	4,45	29,76	46,69	1,75
nanofluid	76,4	76,0	0,40	64,67	64,22	0,45	4,79	4,45	29,89	46,69	1,76
nanofluid	80,6	80,2	0,40	68,87	68,42	0,45	5,10	4,45	30,20	46,69	1,77
nanofluid	82,7	82,3	0,40	70,97	70,52	0,45	5,26	4,45	30,36	46,69	1,77
nanofluid	86,8	86,4	0,40	75,07	74,62	0,45	5,56	4,45	30,66	46,69	1,82
nanofluid	88,9	88,5	0,40	77,17	76,72	0,45	5,72	4,45	30,82	46,69	1,83
nanofluid	91,0	90,6	0,40	79,27	78,82	0,45	5,87	4,45	30,97	46,69	1,83
nanofluid	93,2	92,8	0,40	81,47	81,02	0,45	6,03	4,45	31,13	46,69	1,86
nanofluid	93,2	92,8	0,40	81,47	81,02	0,45	6,03	4,45	31,13	46,69	1,86
nanofluid	93,2	92,8	0,40	81,47	81,02	0,45	6,03	4,45	31,13	46,69	1,86
nanofluid	93,2	92,8	0,40	81,47	81,02	0,45	6,03	4,45	31,13	46,69	1,86
nanofluid	93,2	92,8	0,40	81,47	81,02	0,45	6,03	4,45	31,13	46,69	1,86
nanofluid	93,2	92,8	0,40	81,47	81,02	0,45	6,03	4,45	31,13	46,69	1,86
nanofluid	93,2	92,8	0,40	81,47	81,02	0,45	6,03	4,45	31,13	46,69	1,86
Post-flush water injection after retention time (72 hr)											
water	1,0	1,0	0	-5,54	-5,54	0,00	-	-	-	-	-
water	2,0	2,0	0	-4,54	-4,54	0,00	-	-	-	-	-
water	3,0	3,0	0	-3,54	-3,54	0,00	-	-	-	-	-

water	4,0	4,0	0	-2,54	-2,54	0,00	-	-	-	-	-
water	5,0	5,0	0	-1,54	-1,54	0,00	-	-	-	-	-
water	6,0	6,0	0	-0,54	-0,54	0,00	-	-	-	-	-
water	7,0	6,6	0,4	0,46	0,12	0,34	0,03	3,36	31,16	50,05	4,56
water	8,1	7,5	0,6	1,56	1,00	0,56	0,12	5,54	31,25	52,23	4,89
water	9,1	8,3	0,8	2,56	1,89	0,67	0,19	6,63	31,32	53,32	5,11
water	10,1	9,1	1,0	3,56	2,66	0,90	0,26	8,90	31,39	55,59	5,34
water	11,4	10,3	1,1	4,86	3,85	1,01	0,36	9,99	31,49	56,68	5,58
water	12,2	11,0	1,2	5,66	4,54	1,12	0,42	11,08	31,55	57,77	5,34
water	13,2	11,9	1,3	6,66	5,42	1,24	0,49	12,27	31,62	58,96	4,78
water	14,3	12,8	1,5	7,76	6,30	1,46	0,57	14,44	31,70	61,13	4,56
water	15,4	13,7	1,7	8,86	7,29	1,57	0,66	15,53	31,79	62,22	3,87
water	16,4	14,6	1,8	9,86	8,17	1,69	0,73	16,72	31,86	63,41	3,83
water	17,4	15,5	1,9	10,86	9,06	1,80	0,80	17,80	31,93	64,49	3,81
water	18,6	16,6	2,0	12,06	10,15	1,91	0,89	18,89	32,02	65,58	3,82
water	19,5	17,5	2,0	12,96	11,05	1,91	0,96	18,89	32,09	65,58	3,74
water	20,3	18,2	2,1	13,76	11,74	2,02	1,02	19,98	32,15	66,67	3,51
water	20,5	18,4	2,1	13,96	11,94	2,02	1,03	19,98	32,16	66,67	3,33
water	21,7	19,6	2,1	15,16	13,14	2,02	1,12	19,98	32,25	66,67	3,12

water	22,6	20,4	2,2	16,06	13,93	2,13	1,19	21,07	32,32	67,76	2,99
water	23,6	21,4	2,2	17,06	14,93	2,13	1,26	21,07	32,39	67,76	2,83
water	24,7	22,4	2,3	18,16	15,91	2,25	1,35	22,26	32,48	68,95	2,77
water	26,2	23,8	2,4	19,66	17,30	2,36	1,46	23,34	32,59	70,03	2,77
water	27,3	24,7	2,6	20,76	18,29	2,47	1,54	24,43	32,67	71,12	2,63
water	28,4	25,7	2,7	21,86	19,28	2,58	1,62	25,52	32,75	72,21	2,46
water	29,3	26,5	2,8	22,76	20,06	2,70	1,69	26,71	32,82	73,40	2,46
water	30,4	27,5	2,9	23,86	21,05	2,81	1,77	27,79	32,90	74,48	2,41
water	31,9	29,0	2,9	25,36	22,55	2,81	1,88	27,79	33,01	74,48	2,40
water	33,0	30,1	2,9	26,46	23,65	2,81	1,96	27,79	33,09	74,48	2,40
water	35,1	32,1	3,0	28,56	25,64	2,92	2,12	28,88	33,25	75,57	2,32
water	37,1	34,1	3,0	30,56	27,64	2,92	2,26	28,88	33,39	75,57	2,33
water	39,2	36,1	3,1	32,66	29,63	3,03	2,42	29,97	33,55	76,66	2,34
water	41,4	38,3	3,1	34,86	31,83	3,03	2,58	29,97	33,71	76,66	2,34
water	43,4	40,2	3,2	36,86	33,71	3,15	2,73	31,16	33,86	77,85	2,33
water	45,4	42,2	3,2	38,86	35,71	3,15	2,88	31,16	34,01	77,85	2,31
water	47,5	44,3	3,2	40,96	37,81	3,15	3,03	31,16	34,16	77,85	2,31
water	49,6	46,3	3,3	43,06	39,80	3,26	3,19	32,25	34,32	78,94	2,32
water	51,7	48,4	3,3	45,16	41,90	3,26	3,35	32,25	34,48	78,94	2,32

water	53,7	50,4	3,3	47,16	43,90	3,26	3,49	32,25	34,62	78,94	2,33
water	55,8	52,5	3,3	49,26	46,00	3,26	3,65	32,25	34,78	78,94	2,33
water	57,9	54,6	3,3	51,36	48,10	3,26	3,80	32,25	34,93	78,94	2,33
water	60,0	56,7	3,3	53,46	50,20	3,26	3,96	32,25	35,09	78,94	2,33
water	62,1	58,8	3,3	55,56	52,30	3,26	4,12	32,25	35,25	78,94	2,33
water	64,2	60,7	3,5	57,66	54,39	3,27	4,27	32,34	35,40	79,03	2,33
water	66,2	62,7	3,5	59,66	56,29	3,37	4,42	33,33	35,55	80,02	2,34
water	68,3	64,8	3,5	61,76	58,39	3,37	4,57	33,33	35,70	80,02	2,34
water	70,6	67,1	3,5	64,06	60,69	3,37	4,75	33,33	35,88	80,02	2,33
water	72,4	68,9	3,5	65,86	62,49	3,37	4,88	33,33	36,01	80,02	2,34
water	76,6	73,1	3,5	70,06	66,69	3,37	5,19	33,33	36,32	80,02	2,39
water	81,8	78,3	3,5	75,26	71,89	3,37	5,57	33,33	36,70	80,02	2,38
water	85,1	81,6	3,5	78,56	75,19	3,37	5,82	33,33	36,95	80,02	2,37
water	87,2	83,6	3,6	80,66	77,18	3,48	5,97	34,42	37,10	81,11	2,38
water	90,1	86,5	3,6	83,56	80,08	3,48	6,19	34,42	37,32	81,11	2,36
water	93,2	89,6	3,6	86,66	83,18	3,48	6,42	34,42	37,55	81,11	2,33
water	98,2	94,6	3,6	91,66	88,18	3,48	6,79	34,42	37,92	81,11	2,36
water	105,7	102,1	3,6	99,16	95,68	3,48	7,35	34,42	38,48	81,11	2,37
water	114,0	110,4	3,6	107,46	103,98	3,48	7,96	34,42	39,09	81,11	2,38

water	121,2	117,6	3,6	114,66	111,18	3,48	8,49	34,42	39,62	81,11	2,36
water	129,6	126,0	3,6	123,06	119,58	3,48	9,12	34,42	40,25	81,11	2,36
water	138,9	135,3	3,6	132,36	128,88	3,48	9,80	34,42	40,93	81,11	2,39
water	146,2	142,6	3,6	139,66	136,18	3,48	10,35	34,42	41,48	81,11	2,39
water	156,2	152,6	3,6	149,66	146,18	3,48	11,09	34,42	42,22	81,11	2,39
water	170,0	166,4	3,6	163,46	159,98	3,48	12,11	34,42	43,24	81,11	2,39
water	186,7	183,1	3,6	180,16	176,68	3,48	13,35	34,42	44,48	81,11	2,39
water	200,2	196,6	3,6	193,66	190,18	3,48	14,35	34,42	45,48	81,11	2,39

*Volumes after being corrected for total dead vol. =11.73 (for first and second step)

**Volumes after being corrected for total dead vol. =6.54 (for third step)

In Table S6, total injected vol (PV) and oil produced from OOIP were calculated using Eq. 4 and Eq. 5.

$$\text{Total injected vol (PV)} = \frac{\text{Total injected vol (cc)}}{\text{PV}} \qquad (4)$$

$$\text{Oil produced from OOIP \%} = \frac{\text{Oil produced (cc)}}{\text{OOIP}} \times 100 \qquad (5)$$

5. Spontaneous imbibition in Amott cell calculations

In this section, we fully described how to prepare one core plug sample (i.e for core A in Table S2) for Amott cell experiments. The cleaned core sample was initially saturated with formation water in the vacuum set up under pressure for 24 hours (step 1; section 3.1; PV=15.08). The core plug was flooded with formation water to calculate absolute permeability (step 2; section 3.2; k=28.32 md), and then replaced by flooding with the crude oil until irreducible water saturation was reached (step 3; section 3.3; Swi=29.77%; OOIP-1=10.59 cc). Finally, the core sample was placed in a pressurized vessel containing crude oil at 750 psi and 90 °C for 21 days (step 4; section 3.4). Then the core was placed in a core-flood apparatus and treated with fluid injection to alter the wetting property from oil-wet to an intermediate state (step 5; section 3.5). We performed three runs, 500-ANPs nanofluid flooding until no oil produced (Run#1), a particular rest time for fluid effect (72 hr) (Run#2) and finally water injection until no oil produced (Run#3). The following results were obtained:

Oil produced in run #1: 5.28 cc

Oil produced in run #3: 4.13 cc

Remained oil inside core: 10.59- (5.28+4.13) =1.18 cc

Then, we saturated treated-sample with crude oil again at room temperature to obtain OOIP-2 of core again. OOIP-2 was calculated using Eq. 6.

$$OOIP - 2 = \frac{w_1 - w_2}{d_{oil}} + v_{ro} \qquad (6)$$

Where:

W_1: weight of core before flooding with crude oil (gr)

W_2: weight of core after flooding with crude oil (gr)

d_{oil}: density of crude oil at 25˚C; 0.85 g/cm^3

v_{ro}: remained oil inside the core after coreflooding test (cc); 1.18 cc

OOIP-2 was obtained 5.8 cc fore core A. In Figure 3, recovery from OOIP was calculated using Eq. 7.

$$\text{Recovery from OOIP} - 2\,\% = \frac{\text{Oil produced (cc)}}{\text{OOIP} - 2} \times 100 \qquad (7)$$

Supporting Figures has been cited in main manuscript:

Figure S1. XRD Patterns of (a) α- Al_2O_3 quantum dots (AQDs), (b) γ-Al_2O_3 nanoparticles (ANPs)

Figure S2. Fluorescence property of α-Al_2O_3 quantum dots (AQDs) sample.

Figure S3. Adsorption isotherms of citric acid on surface of (a) α-Al_2O_3 quantum dots (AQDs) and (b) γ-Al_2O_3 nanoparticles (ANPs) at pH=7.1

Figure S4. The FT-IR Spectrum of citric acid adsorption on alumina at pH=7.1 a) γ-Al_2O_3 nanoparticles (ANPs) b) α-Al_2O_3 quantum dots (AQDs)

Figure S5. Calibration plots for Al_2O_3 stability as a function of particle concentration from UV-vis analysis at 25˚C.

Figure S6. Colloidal stability of citrate- alumina particles (AQDs and ANPs) in SWP, T=25˚C and 90˚C, retention time=24 h from UV-vis analysis.

Figure S7. Colloidal stability of PE-citrate-alumina particles (AQDs and ANPs) in SWP, T=25˚C and 90˚C, retention time=24 h from UV-vis analysis.

Figure S8. D_H value distribution of alumina particles in various brines after 30 days; T=90˚C a)500-PE-cit-AQDs b) 1000-PE-cit-AQDs c) 500-PE-cit-ANPs nanofluids

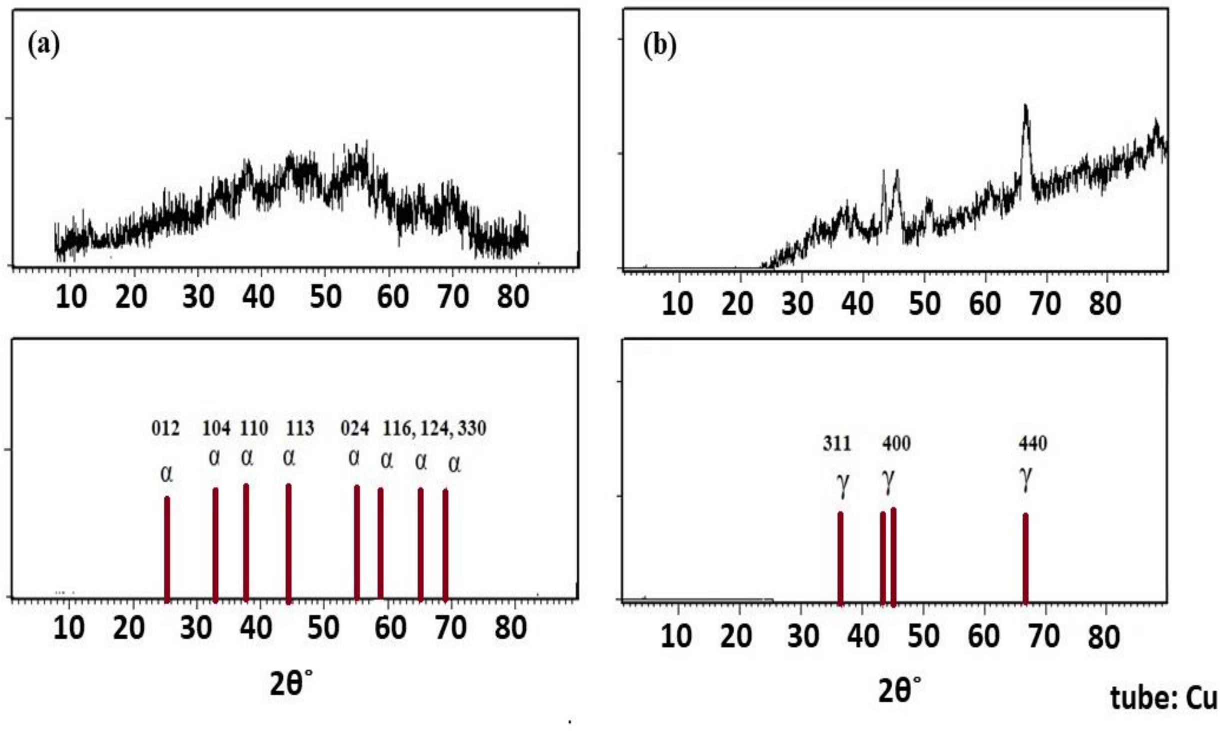

Figure.S1. XRD Patterns of (a) α- Al_2O_3 quantum dots (AQDs), (b) γ-Al_2O_3 nanoparticles (ANPs)

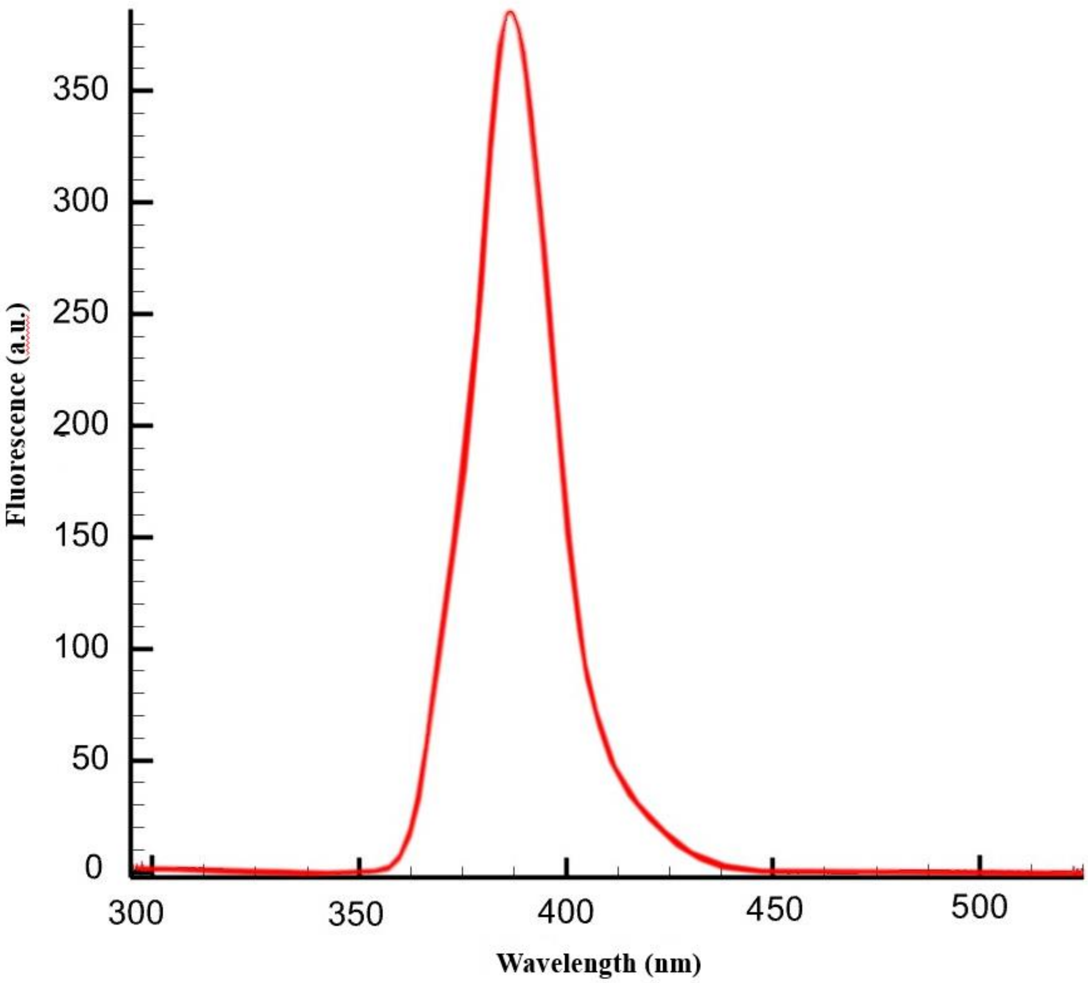

Figure S2. Fluorescence property of α-Al_2O_3 quantum dots (AQDs) sample.

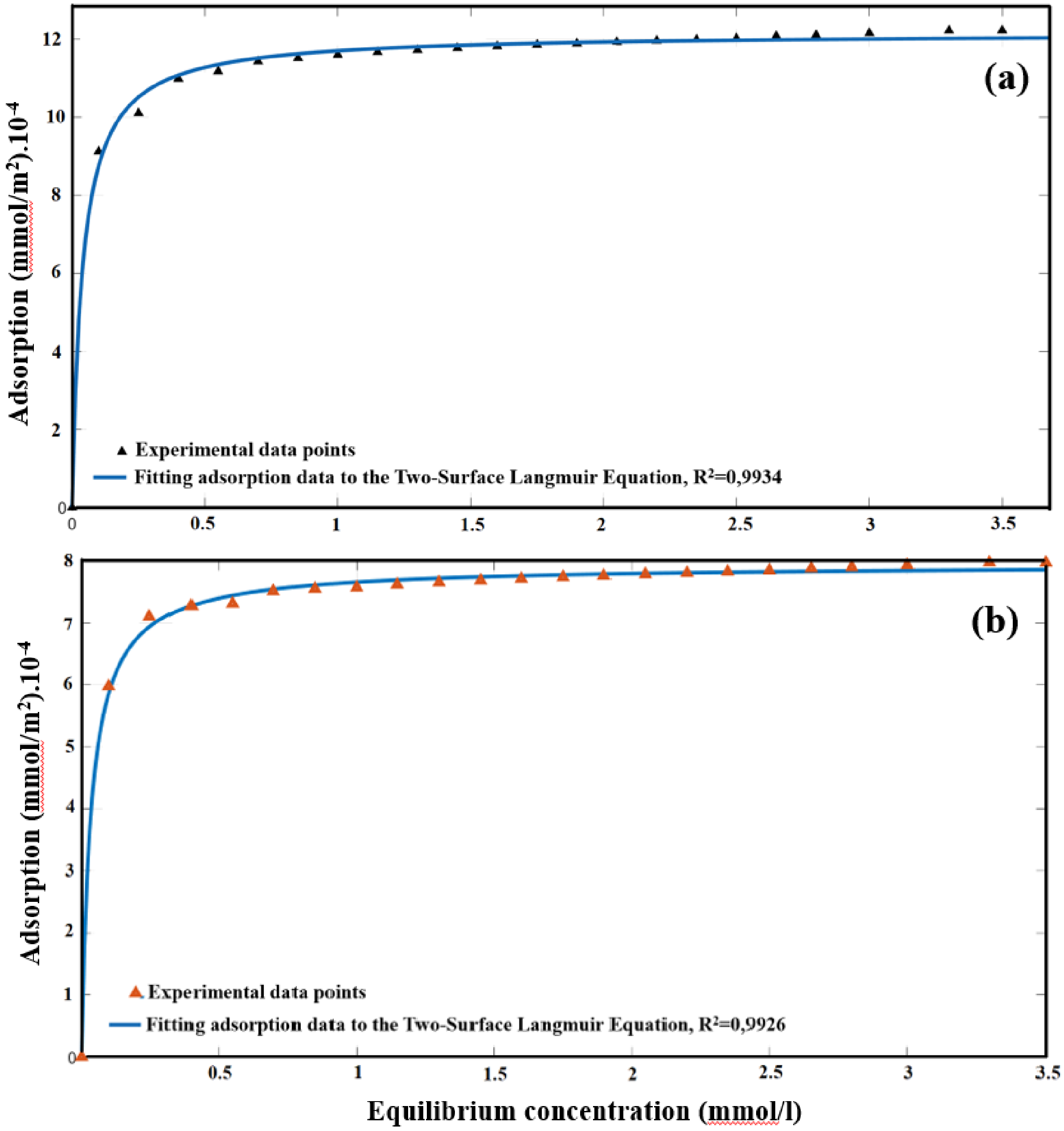

Figure S3. Adsorption isotherms of citric acid on surface of (a) α-Al_2O_3 quantum dots (AQDs) and (b) γ-Al_2O_3 nanoparticles (ANPs) at pH=7.1

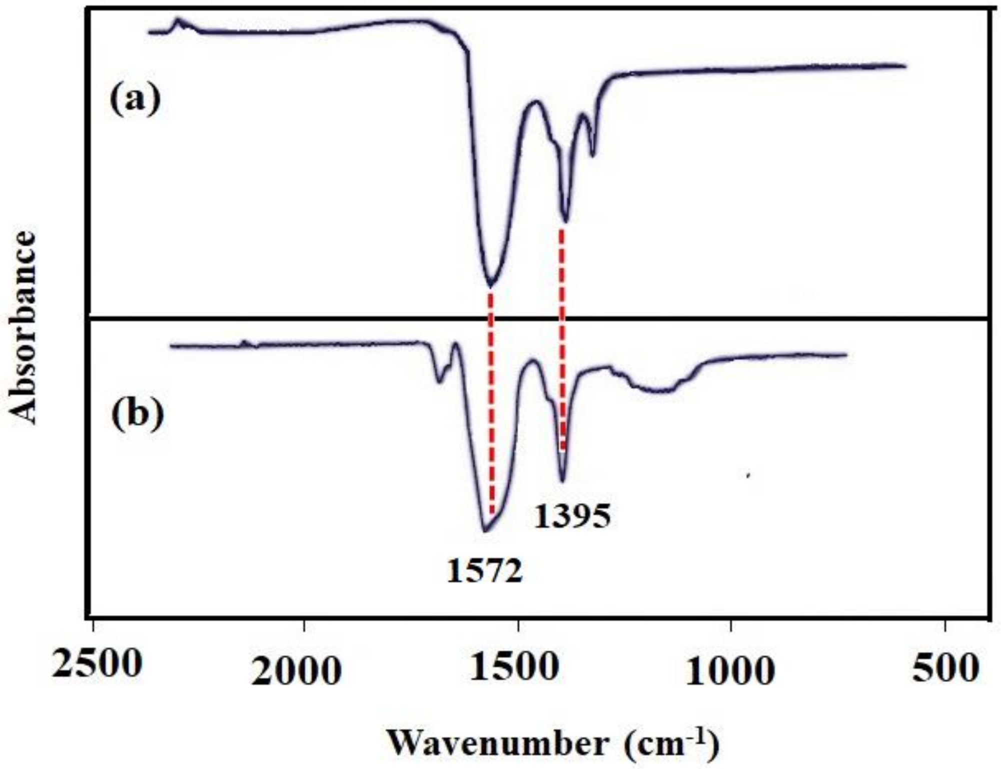

Figure S4. The FT-IR Spectrum of citric acid adsorption on alumina surface at pH=7.1 a) cit-γ-Al_2O_3 nanoparticles (ANPs) b) cit-α-Al_2O_3 quantum dots (AQDs)

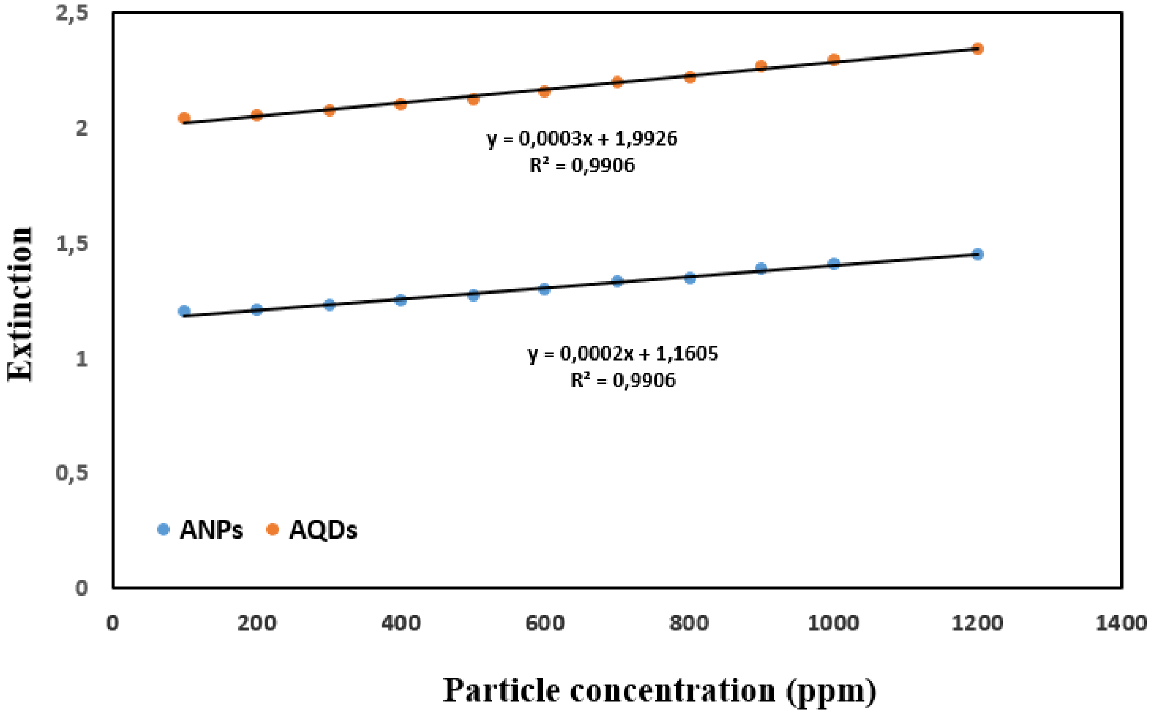

Figure S5. Calibration plots for citrate-coated-Al_2O_3 stability as a function of particle concentration from UV-vis analysis at 25˚C.

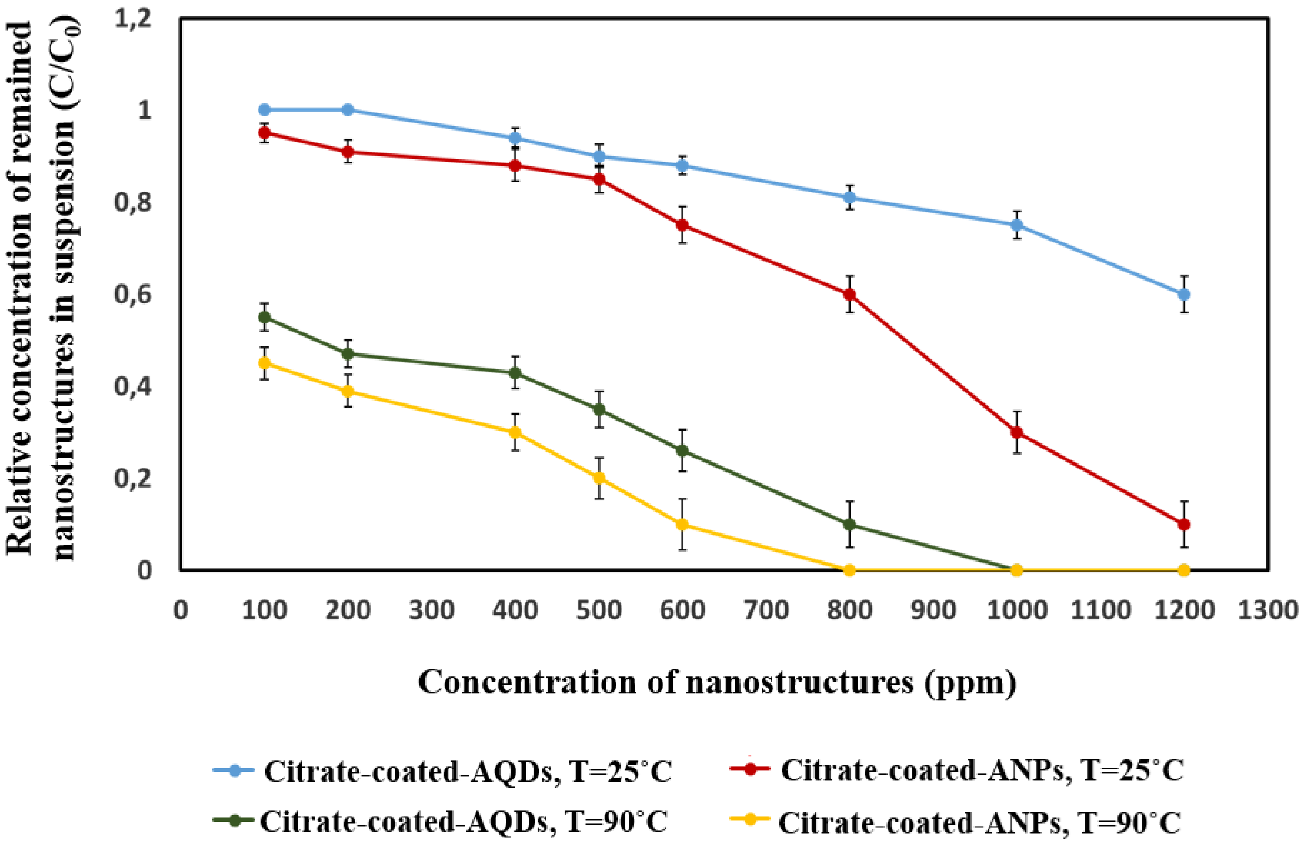

Figure S6. Colloidal stability of citrate- alumina particles (AQDs and ANPs) in SWP, T=25˚C and 90˚C, retention time=24 h from UV-vis analysis.

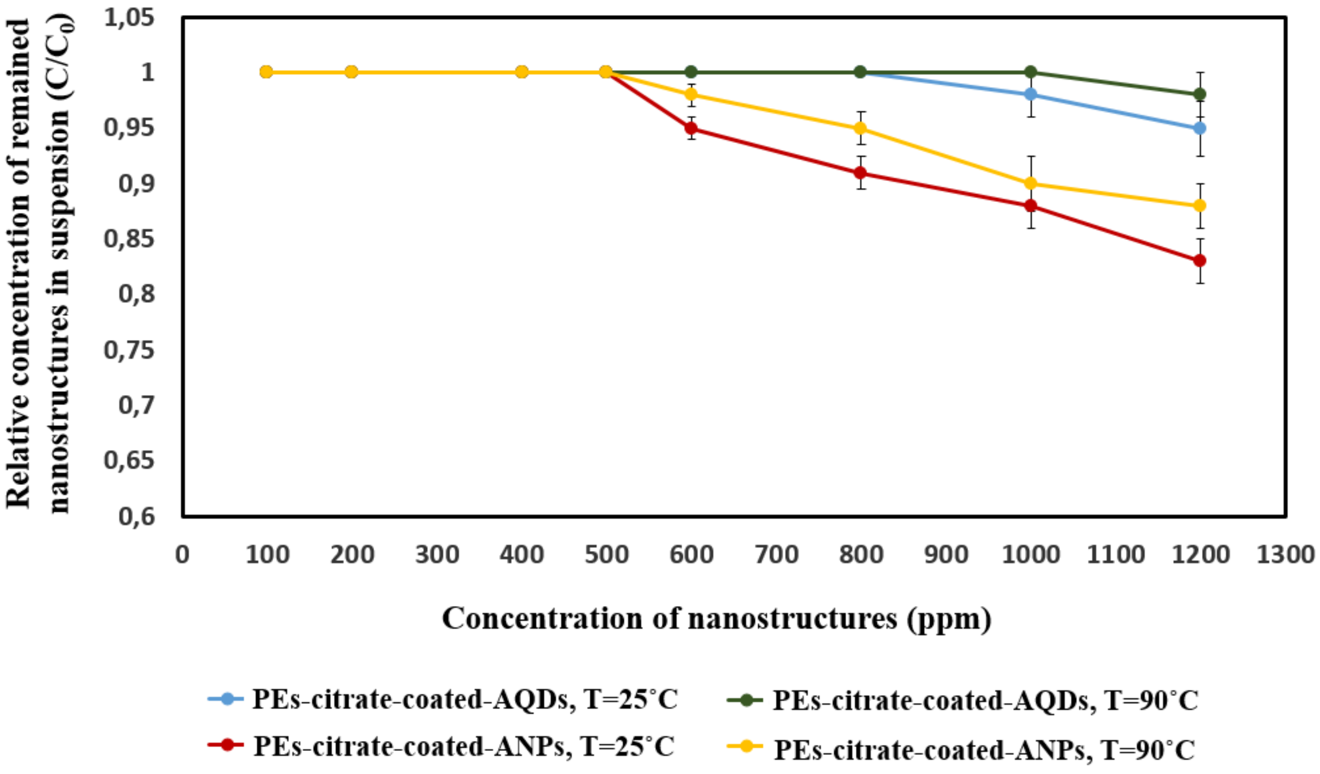

Figure S7. Colloidal stability of PE-citrate-alumina particles (AQDs and ANPs) in SWP, T=25˚C and 90˚C, retention time=24 h from UV-vis analysis.

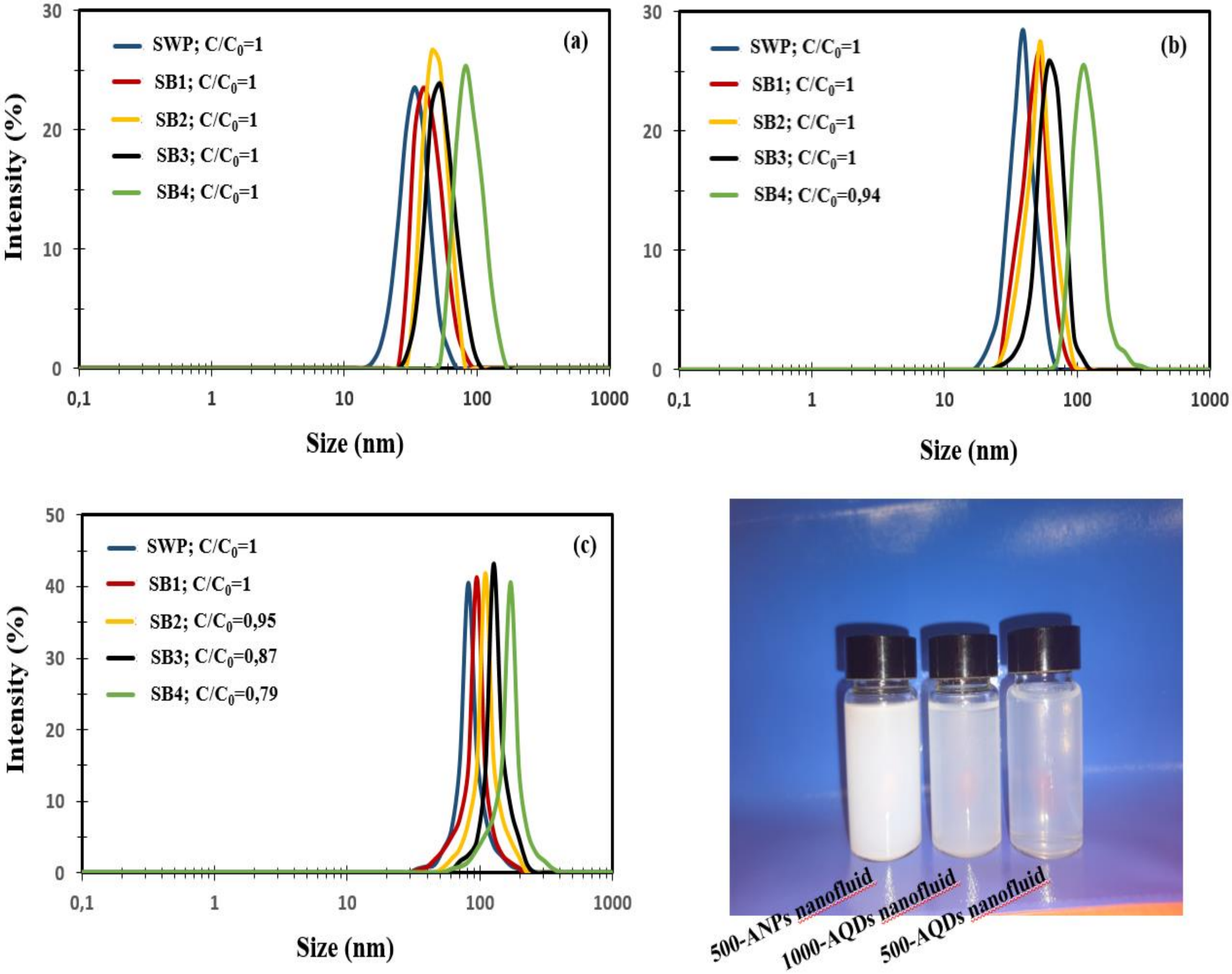

Figure S8. D_H-value distribution of alumina particles in various brines after 30 days; T=90˚C a)500-PE-cit-AQDs b) 1000-PE-cit-AQDs c) 500-PE-cit-ANPs nanofluids.

Supporting Tables has been cited in main manuscript:

Table S1. Ion compositions of formation water, sea water and synthetic brines

Table S2. Physical properties and description of core samples

Table S3: The adsorptive properties of alumina nanostructures from ASAP data

Table S1. Ion compositions of formation water, sea water and synthetic brines

Ionic	Formation water, FW (ppm)	Persian gulf sea water, SWP (ppm)	Synthetic brine, SB1 (ppm)	Synthetic brine, SB2 (ppm)	Synthetic brine, SB3 (ppm)	Synthetic brine, SB4 (ppm)
Calcium	20000	520	15000	25000	35000	45000
Barium	3000	0	0	0	0	0
Strontium	490	5	0	0	0	0
Sodium	72000	13000	15000	20000	25000	30000
Potassium	3700	400	0	0	0	0
Magnesium	2500	1400	0	0	0	0
Iron	3000	0	0	0	0	0
Bicarbonate	150	160	0	0	0	0
Sulfate	380	3300	0	0	0	0
Chloride	157000	23000	49679.65	75085.53	100491.40	125897.30
Salinity	262220	41780	79679.65	120085.50	160491.40	200897.30

Table S2. Physical properties and description of core samples

	Physical properties				Spontaneous imbibition tests		Core displacement tests		Formation damage tests		
Core ID	D (cm)	L (cm)	OOIP (cc)	Liquid absolute perm (md)	Type	Imbibing fluid	Type	Injection Scenario	Type	Injection Scenario	S_{wi} %
II	3.80	6.40	11.23	27.41	Initial oil-wet; treated with SWP	Brine	-	-	-	-	25.45
A	3.80	6.40	10.59	28.26	Initial oil-wet; treated with 500-PE-cit-ANPs nanofluid	Brine	-	-	-	-	29.77
B	3.80	6.40	7.95	30.21	Initial oil-wet; treated with 500-PE-cit-AQDs nanofluid	Brine	-	-	-	-	31.53
C	3.80	6.40	9.42	25.45	Initial oil-wet; treated with 1000-PE-cit-AQDs nanofluid	Brine	-	-	-	-	24.65
D	3.80	6.40	10.11	16.51	-	-	Initial oil-wet	Brine/500-PE-cit-ANPs/Brine	-	-	25.16
E	3.80	6.40	9.42	15.31	-	-	Initial oil-wet	Brine/500-PE-cit-AQDs/Brine	--	-	29.45
F	3.80	6.40	11.22	18.16	-	-	Initial oil-wet	Brine/1000-PE-cit-AQDs/Brine	-	-	26.72
D’	3.80	6.40		15.51	-	-	-	-	Initial water-wet	Brine/Brine	-
E’	3.80	6.40		15.12	-	-	-	-	Initial water-wet	Brine/Brine	-
F’	3.80	6.40		17.65	-	-	-	-	Initial water-wet	Brine/Brine	-
D”	3.80	6.40		14.90	-	-	-	-	Initial water-wet	500-PE-cit-ANPs/Brine	-
E”	3.80	6.40		14.85	-	-	-	-	Initial water-wet	500-PE-cit-AQDs/Brine	-
F”	3.80	6.40		17.11	-	-	-	-	Initial water-wet	1000-PE-cit-AQDs/Brine	-

- Cores D’, E’ and F’ are the same as cores D, E and F that restored by implementing cleaning procedures again.

- Cores D”, E” and F” are the same as cores D’, E’ and F’ that restored by implementing cleaning procedures again.

Table S3: The adsorptive properties of alumina nanostructures from ASAP data.

Samples	S_{Total} (m^2/g)	V_{total} (cm^3/g)
α-Al_2O_3(AQDs)	112.43	0.29
γ-Al_2O_3(AQDs)	90.25	0.12
